# Proteomic and Structural Manifestations of Cardiomyopathy in Rat Models of Obesity and Weight Loss

**DOI:** 10.3389/fendo.2021.568197

**Published:** 2021-02-24

**Authors:** Arkadiusz D. Liśkiewicz, Łukasz Marczak, Katarzyna Bogus, Daniela Liśkiewicz, Marta Przybyła, Joanna Lewin-Kowalik

**Affiliations:** ^1^ Department of Physiology, Faculty of Medical Sciences in Katowice, Medical University of Silesia, Katowice, Poland; ^2^ Laboratory of Molecular Biology, Institute of Physiotherapy and Health Sciences, The Jerzy Kukuczka Academy of Physical Education, Katowice, Poland; ^3^ Institute of Bioorganic Chemistry, Polish Academy of Sciences, Poznan, Poland; ^4^ Department of Histology, Faculty of Medical Sciences in Katowice, Medical University of Silesia, Katowice, Poland; ^5^ Department for Experimental Medicine, Faculty of Medical Sciences in Katowice, Medical University of Silesia, Katowice, Poland

**Keywords:** cafeteria diet, caloric restriction, cardiac fibrosis, developmental obesity, heart proteomics, obesity cardiomyopathy, plasma proteomics, weight loss

## Abstract

Obesity cardiomyopathy increases the risk of heart failure and death. Obesity is curable, leading to the restoration of the heart phenotype, but it is not clear if there are any after-effects of obesity present after weight loss. We characterize the proteomic landscape of obesity cardiomyopathy with an evaluation of whether the cardiac phenotype is still shaped after weight loss. Cardiomyopathy was validated by cardiac hypertrophy, fibrosis, oversized myocytes, and mTOR upregulation in a rat model of cafeteria diet-induced developmental obesity. By global proteomic techniques (LC-MS/MS) a plethora of molecular changes was observed in the heart and circulation of obese animals, suggesting abnormal utilization of metabolic substrates. This was confirmed by increased levels of cardiac ACSL-1, a key enzyme for fatty acid degradation and decreased GLUT-1, a glucose transporter in obese rats. Calorie restriction and weight loss led to the normalization of the heart’s size, but fibrosis was still excessive. The proteomic compositions of cardiac tissue and plasma were different after weight loss as compared to control. In addition to morphological consequences, obesity cardiomyopathy involves many proteomic changes. Weight loss provides for a partial repair of the heart’s architecture, but the trace of fibrotic deposition and proteomic alterations may occur.

## Introduction

According to International Statistical Classification of Diseases and Related Health Problems (10th revision), obesity is defined as a disorder characterized by an abnormally high, unhealthy amount of body fat and is listed as E66 subgroup of “endocrine, nutritional and metabolic diseases.” This pandemic disease has many severe consequences including an increased risk of death, morbidity, and accelerated aging (1) due to the many associated systemic changes. The disease is widespread globally across all age groups (2, 3). In the United States, one in five children are obese and the scale of the problem is well illustrated by reported cases of obesity among children. Obesity symptoms are potentially curable by losing weight due to reducing calorie intake and increasing energy expenditure. Other methods that are effective for weight loss include medication and surgery (4). Reversing obesity is essential for lifespan and healthspan because it affects all the systems of the human body, especially contributing to the development of cardiovascular diseases (5). Obesity and the obesogenic diet are leading causes of hypertension (6). A pressure overload leads to pathological cardiac hypertrophy and overgrown myocytes enmeshed within an abundant network of the extracellular matrix (ECM) is the hallmark of obesity cardiomyopathy and left ventricle (LV) remodeling (7). Although pressure overload is the predominant inductor of LV hypertrophy (8), the hypertrophied heart develops independently of hypertension during obesity (9), suggesting that other mechanisms may also contribute to the cardiac overgrowth. The hypertrophy of cardiomyocytes with cardiac fibrosis and remodeling associated with the obesogenic diet and weight gain may be a consequence of the intensification of intracellular anabolic processes and sustention of a pro-inflammatory status (10, 11). Normally cardiac fibroblasts make up about 15% of non-myocyte cardiac cell type (12) and support cardiomyocytes by producing ECM and by regulating the proliferation and migration of other cardiac cells (13). Thus, fibroblasts play an important role in cardiac repair. However, in certain circumstances the excessive proliferation and differentiation of fibroblasts lead to the increased deposition of fibrotic content (fibrosis) and consequently to heart failure (14).

Weight reduction and weight loss maintenance is capable of reversing many of the alterations in cardiac performance and morphology associated with obesity (15) and is essential in avoiding heart failure related to obesity cardiomyopathy (16–18). However, more detailed investigations are needed in order to identify the potential changes that are persistent after weight loss and to find treatment options.

This study uses animal models to demonstrate how developmental obesity affects proteome in obesity cardiomyopathy and identifies cardiac changes that are present after weight normalization.

## Materials and Methods

### Experimental Design

All animals were provided by the Animal House of the Department for Experimental Medicine, Medical University of Silesia, Katowice, Poland and were treated in accordance to Directive 2010/63/EU for animal experiments using the protocols approved and monitored by the Local Ethics Committee for Animal Experimentation in Katowice (approved protocol number 22/2017). Animals were housed 4–5 per cage in a climate-controlled room (22 ± 2°C, relative humidity: 55 ± 10%) with a 12 h:12 h light/dark cycle starting at 07:00 a.m.

Young (postnatal day 28) Long Evans male rats were used in two independent experiments. In Exp.1, animals were fed ad libitum with standard chow (control to obese cohort, ContO, n=16) or the same chow supplemented with cafeteria diet foods (Obese group, n=16; refer to ‘Obesogenic rodent’s diet’ section for more details) to gain weight. After 12 weeks the animals were euthanized by decapitation to collect blood and heart samples.

In Exp. 2, animals from the AWL group (After Weight Loss, n=16) were treated in the same manner as in Exp. 1 to develop obesity within 12 weeks. After this period the obese rats were subjected to calorie restriction (weight-loss-phase) for 6 weeks. During this phase the rats from AWL group were subjected only to standard rodent chow with an 80% calorie restriction (CR) for the first two weeks, which was then reduced to 70% until the end of this period. The 100% of caloric needs was established empirically – the isocaloric serving was established when the amount of a given chow ensured that rat’s body weight has been stable (without gaining or losing weight) for 24 h. The control animals to AWL group (ContA, n=16) were fed ad libitum by standard chow and water during the weight-gaining-phase of Exp. 2. During the weight-loss-phase ContA animals received controlled portions of the food to stop subtle weight gain. The amount of food was established empirically for every cage and 20 g (~70 kcal) of standard chow/rat/day determined the criteria.

After the weight loss the stabilization phase was introduced during which the rats from AWL and ContA groups received an isocaloric amount of food (20 g of standard chow/rat/day) for four weeks to equalize the calorie intake among control and experimental groups (please refer to discussion section for detailed explanation). Drinking water was provided ad libitum during all phases of Exp. 2.

### Obesogenic Rodent’s Diet

The standard diet energy content (3.57 kcal/g) came from 67% carbohydrates, 25% proteins and 8% fats. To mimic the human obesogenic diet, the animals were fed with commercially available human snack foods. The components of the cafeteria diet were chosen to reflect the variety, palatability and energy density of the human diet. The food was supplied daily. The animals received one of two sets of snacks interchangeably. These were given to the animals on alternate days as one diversified diet. Set 1 contained: candy bar (Mars; Mars Inc.), crackers (Lajkonik Snacks), and kabanos (dry sausage made of pork; Tarczyński). Set 2 contained: candy bar (Bounty; Mars Inc.), potato chips (Lays Salt; PepsiCo), and Tilsit cheese (Hochland). The average caloric density of these two dietary sets was 4.84 kcal/g with the following caloric profile: carbohydrates 33.2%, fat 33.1%, and proteins 16.6%. The animals in this group received clean water and a sweet beverage – 10% sucrose solution in a second container. By exposing rats to a variety of highly palatable foods high in fat and sugar, the cafeteria diet protocol described here was previously proved to provide a reliable and robust model of the so-called “western diet” eaten by many people (19–21).

### Tissue Sampling

Six hours prior to insulin tolerance and glucose loading tests the rats were fasted (tested animals from Exp.1 were fed by standard chow and water for 24 h before fasting), than insulin (1 U/kg in Exp.1; 0.75 U/kg in Exp.2; ActiRapid, Novo Nordisc) or glucose (2 g/kg) were injected in i.p. injections. Blood was collected from the tip of tail at the appropriate time intervals to measure glucose levels (CardioCheck Professional). The rats used for insulin tolerance and glucose loading tests (n=5 in each group of Exp. 1 and Exp. 2) did not undergo further evaluation in this study.

For the histological examination of the morphology of cardiomyocytes, six hearts per group collected during Exp. 1 and Exp. 2 were gently squeezed to remove excess blood, then weighed and immersed in a 10% formalin solution in PBS (pH=7.2). For proteomic examination, fresh pieces of LV were collected from another animals (n=5 per group in Exp. 1). In Exp. 2, we increased the number of samples used for proteomics by collecting additional pieces of LV form the hearts of animals intended for histology (from three rats per group) since this kind of analysis has not been done before in a weight loss model (n=8 per group).

After decapitation ~2 ml of trunk blood was collected for serum and plasma samples. For the serum collection, the blood was allowed to clot by leaving it for 30 min on ice in Eppendorf tubes. The samples were then centrifuged (2,000× g for 15 min, 4C), and the serum was pipetted and stored at −80 °C. Serum cholesterol and triglyceride levels were measured in the sera using a Mindray BS-200 Chemistry Analyzer (Shenzhen Mindray Bio-Medical Electronics Co.).

In order to obtain plasma (used for proteomic profiling), 1 ml of blood was collected into 1.5 ml EDTA coated Eppendorf tubes containing 10 ul of 0.5 M EDTA and immediately centrifuged (1,300× g for 10 min, 4C). 99 ul of plasma was pipetted to fresh tubes containing 1 ul of a protease inhibitor cocktail (#P8340, Sigma Aldrich) (22).

### Histological Examination of the Heart

The LV were dissected from formalin-fixed hearts. The tissues were dehydrated with graded concentrations of alcohol and embedded in paraffin. 5 μm paraffin slices from each tissue sample were stained with (i) Hematoxylin and Eosin (H&E) or (ii) Masson’s trichrome stain.

To calculate the cross-sectional area of the cardiomyocyte, the sections stained with H&E were photographed (with 40x objective) using a Nikon light microscope (Nikon ECLIPSE E600) with an Olympus Camera (Olympus DP 26). The individual cell surface area was measured by a blinded observer using cellSens Entry Imaging Software (Olympus). Twenty five cell surface areas were counted per each animal (n=3–4), and the average value was used for analysis.

Masson’s stain was used to investigate LV morphology and perivascular/interstitial fibrotic changes. The sections were photographed (with 40x objective) six times each for the following: LV area without visible vessels (interstitial zone) and area with visible vessels (perivascular zone) (23). The photos were subjected to deconvolution in the NIH Fiji program (24), than the areas of blue (connective tissue) or red (myocytes) channels were automatically counted. The data is expressed as the amount of connective tissue relative to the total area (connective tissue+myocytes) and expressed as a %.

### Proteomic Profiling

#### Protein Extraction

The isolated tissues were lysed in a buffer with 1 M triethylammonium bicarbonate (TEAB) and 0.1% sodium dodecyl sulfate (SDS) and automatically homogenized using a Precellys 24 homogenizer (Bertin Technologies) in 0.5-ml tubes pre-filled with ceramic (zirconium oxide) beads (Bertin Technologies). Next, the material was subjected to a threefold cycle of freezing and thawing. Then, the tissue in the buffer was sonicated in a bath for three 1-min cycles on ice and homogenized again using the Precellys 24 instrument. The protein concentration was measured using Pierce BCA protein assay kit (Thermo Fisher Scientific) in the isolated protein fraction according to the manufacturer’s instructions.

#### In-Solution Digestion

Ten-microgram aliquots of the proteins were diluted with 15 µl of 50 mM NH4HCO3 and reduced with 5.6 mM DTT for 5 min at 95°C. The samples were then alkylated with 5 mM iodoacetamide for 20 min in the dark at RT. The proteins were digested with 0.2 µg of sequencing-grade trypsin (Promega) overnight at 37°C.

#### Liquid Chromatography-Tandem Mass Spectrometry (LC-MS/MS) Analysis of the Proteins

The analysis was performed with the use of the Dionex UltiMate 3000 RSLC nanoLC System connected to the Q Exactive Orbitrap mass spectrometer (Thermo Fisher Scientific). The peptides derived from the in-solution digestion were separated on a reverse phase Acclaim PepMap RSLC nanoViper C18 column (75 µm × 25 cm, 2 µm granulation) using an acetonitrile gradient (from 4% to 60%, in 0.1% formic acid) at 30°C and a flow rate of 300 nL/min (for 230 min). The spectrometer was operated in data-dependent MS/MS mode with survey scans acquired at a resolution of 70,000 at m/z 200 in MS mode, and 17,500 at m/z 200 in MS2 mode. The spectra were recorded in the scanning range of 300–2,000 m/z in the positive ion mode. Higher energy collisional dissociation (HCD) ion fragmentation was performed with normalized collision energies set to 27.

#### Protein Data Analysis

Protein identification was performed using the Swiss-Prot rat database with a precision tolerance set to 10 ppm for peptide masses and 0.08 Da for fragment ion masses. All raw data obtained for each dataset was imported into MaxQuant 1.5.3.30 version for protein identification and quantification. Protein was considered as positively identified if at least two peptides per protein were found by the Andromeda search engine, and a peptide score reached the significance threshold FDR=0.01.

The obtained data was exported to Perseus ver. 1.5.3.2 software (part of the MaxQuant package). The numeric data was transformed to the logarithmic scale and each sample was annotated with its group affiliation. Next, the data was filtered based on valid values – proteins, which had valid values in 70% of samples in at least one group were kept in the table. Student’s t-test was performed on the analyzed sample data with permutation-based FDR 0.05 used for truncation and the resulting list of differentiating proteins was transformed using a Z-score algorithm for the hierarchical clustering of data.

### Immunoblotting

For immunoblotting, the samples were separated on 4%–15% Stain-Free Gel (Bio-Rad) and transferred onto PVDF membranes. The membranes were blocked for 1 h at room temperature in Casein Blocking Buffer (Sigma-Aldrich) or 5% BCA, and incubated overnight at 4°C (TBST+adequate blocking buffer) with primary antibodies produced in rabbits: anti-phospho AMPKα (Thr172) (Cell Signaling, #2535), anti-phospho mTOR (Thr2446 and Ser2448) (#15-105, MERK Millipore), anti-GLUT-4 (#PA1-1065, Invitrogen), anti-GLUT-1 (#ab652, Abcam), and anti-ACSL-1 (#PA5-17136, Invitrogen). After washing, the membranes were incubated with secondary donkey anti-rabbit IgG antibody (Abcam, ab205722). The immunoblots were visualized by means of Clarity Western ECL Blotting Substrates (Bio-Rad) and detected with the ChemiDoc™ Touch Imaging System (Bio-Rad). The targeted proteins were quantified with ImageLab Software 6.0.1 (Bio-Rad). The results were normalized to the total protein content in the gel (Stain-free technology, Bio-Rad).

### Statistical Analysis

Statistical analysis was performed using GraphPad Prism 8.01 software (GraphPad Software Inc.). Depending on the data distribution (as evaluated by the Shapiro-Wilk normality test), either the two tailed Student’s t-test or U-Mann-Whitney test were used for the estimation of significant differences between the two subject groups. In the case of repeating measurements, two-way ANOVA with Sidak’s multiple comparison test was applied. In all tests, significance was considered when p<0.05. Data on the graphs are presented as mean and data points (sample size), unless otherwise stated.

Gene ontology (GO)-term enrichment analysis was performed with the DAVID functional annotation tool (25, 26). The complete list of Rattus norvegicus proteins detected in mass spectrometry-based proteomics was used for the background and the GO term subcategories “GOTERM_BP_DIRECT” “GOTERM_CC_DIRECT” and “GOTERM_MF_DIRECT” “INTACT” were selected for analysis EASE threshold 0.1). A modified Bonferroni correction of p-value was applied to identify the statistically more represented function annotations and GO-process was considered as significant when p<0.05.

## Results

### The Obesity Phenotype Is Associated With Heart Hypertrophy

**Figure 1:**
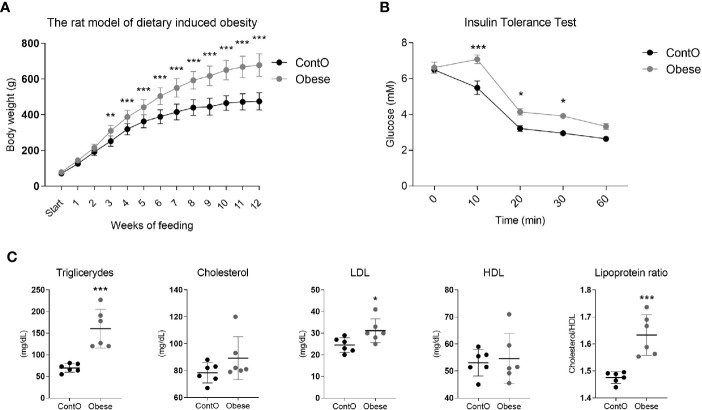
Characteristics of the obesity phenotype in rats fed with a cafeteria diet for 12 weeks. As a consequence of high-calorie feeding, the animals (n=16) gained weight intensively **(A)** and developed insulin resistance (n=5) **(B)** with hyperlipidemia and hypercholesterolemia (n=6) **(C)**. Two-way ANOVA with Sidak’s multiple comparison test **(A, B)** and the Student’s t-test or Mann-Whitney test (cholesterol) were used for analyzing the data (*p<0.05, **p<0.01, ***p<0.001).

**Figure 2:**
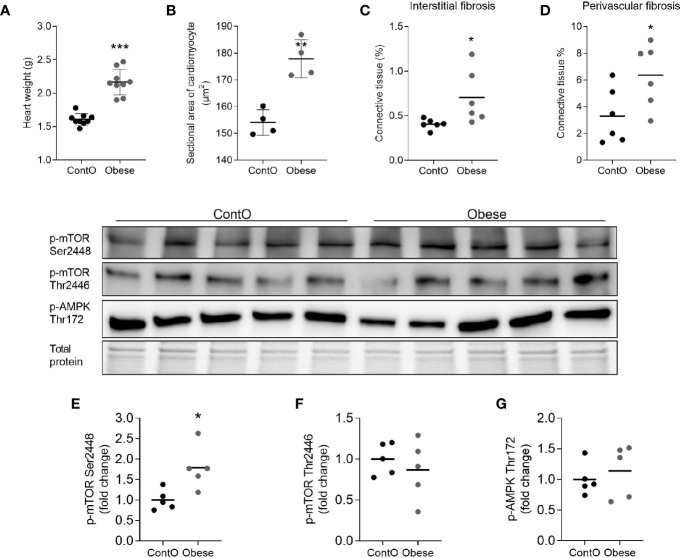
Hallmarks of obesity cardiomyopathy in rats. The hearts of obese animals were heavier (n=9) **(A)** with enlarged myocytes (n=4) **(B)**. The oversized hearts contained higher levels of fibrotic tissue both in the non-vessel **(C)** and vessel containing **(D)** areas (n=6). The expression of phospho-mTOR on ser2448 **(E)** but not on Thr2446 **(F)** was higher in the LV of obese rats, suggesting the up-regulation of this anabolic pathway (n=5). No significant differences were observed with regard to of AMPK activity **(G)**. The Student’s t-test was used for analyzing the data (*p<0.05, **p<0.01, ***p<0.001).

In order to evaluate the influence of developmental obesity on the heart proteomic profile, the rats were fed with a cafeteria diet starting from postnatal day 28 for 12 weeks. Starting with the 3rd week of feeding, these rats gained weight more excessively than control (**Figure 1**). The obesity phenotype resulted in decreased response to insulin measured by ITT (**Figure 1**). An analysis of sera revealed higher triglycerides (p=0.0007) and LDL (p=0.03) concentrations in obese rats (**Figure 1**). These rats also had increased total-cholesterol-to-HDL ratio (p=0.0006, **Figure 1**), which is recognized as a strong cardiovascular risk marker (27). Ten weeks after introduction of diet-induced obesity protocol, the hearts were collected from animals to perform morphometric and proteomic analyses. The hearts of obese rats were about 35% heavier than in control (p<0.0001; **Figure 2**). The morphometric measurement performed in H&E-stained sections of LV revealed increased area of cardiomyocytes in obese animals (p=0.0015) (**Figure 2**). The microscopic evaluation of Masson-Trichrome stained slides revealed a higher amount of connective tissue immersed between the myocytes (**Figure 2**) or localized around the vessels (**Figure 2**). Enhanced phosphorylation of mTOR on Ser2448 was observed in the LV of obese rats (p=0.016) (**Figure 2**), suggesting the up-regulation of this anabolic pathway. On the other hand, the phosphorylation of mTOR on Thr2446 did not differ among groups (**Figure 2**). This mTOR region is phosphorylated during AMPK activation resulting in mTOR pathway inhibition (28). In line with this, we did not observe significant changes in AMPK signaling (**Figure 2**).

### The Proteomic Status of the Cardiac Cells Exhibited a Stronger Shift Toward the Utilization of Fatty Acids in Obese Animals

**Figure 3:**
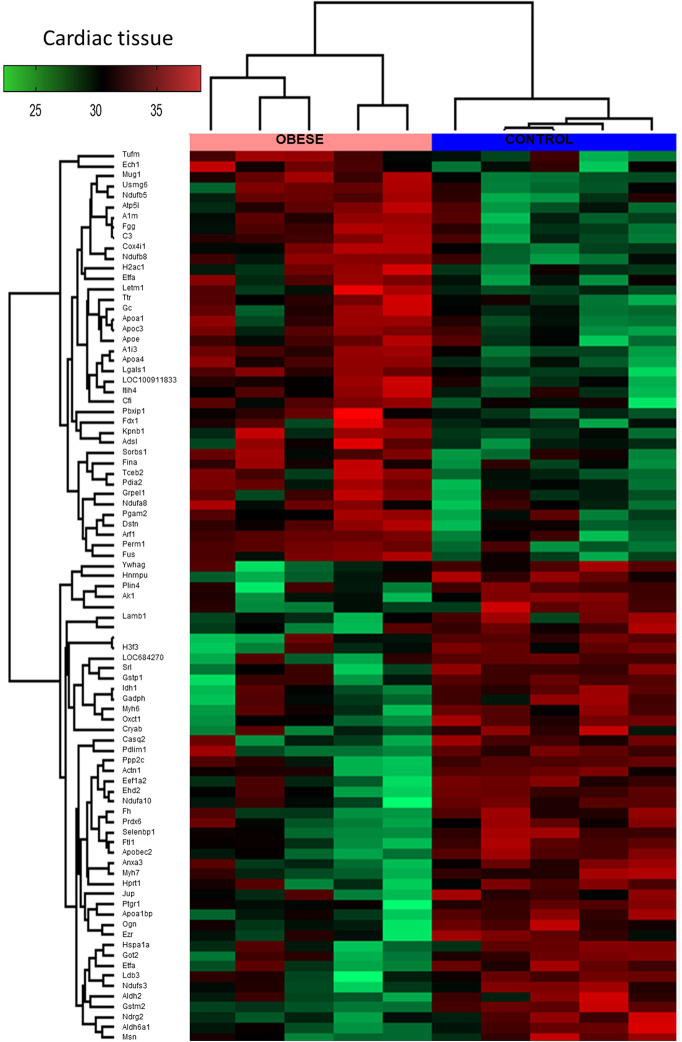
Global proteomic profile of the cardiac muscle of obese rats. The amount of 87 proteins differed in the left ventricle (LV) tissue of obese rats as compared to homogenous samples from control (n=5). Protein expression data are presented as Z-score transformed.

**Figure 4:**
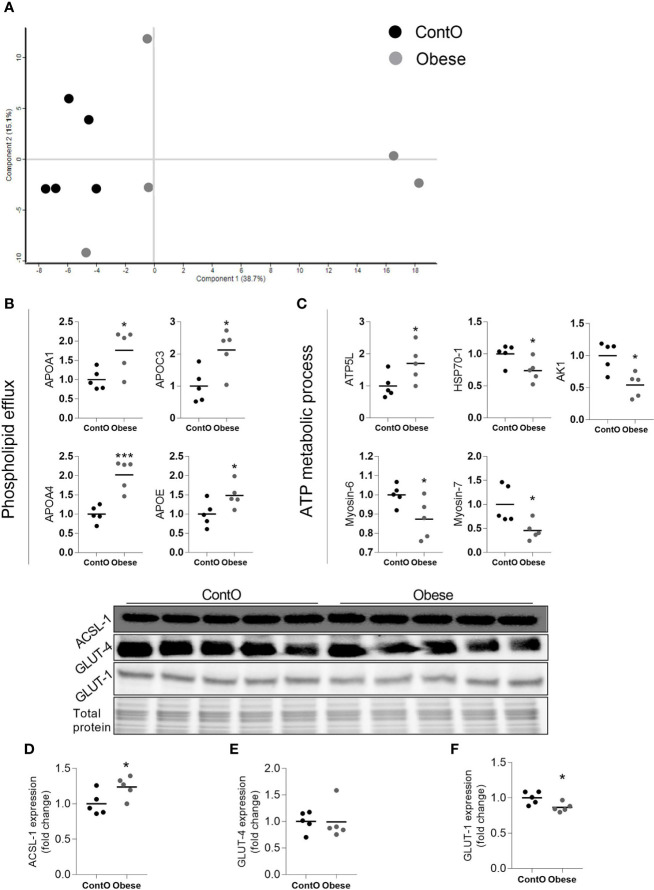
Molecular processes changed in the heart muscle of obese rats (n=5). A scaling plot of individual proteomes revealed the relatively homogenous composition in the control group (ContO) and the more dispersed proteomic phenotype in experimental (obese) animals **(A)**.Two biological processes: **(B)** the directed movement of a phospholipid out of a cell or organelle (p=0.024), and **(C)** the chemical reactions and pathways involving ATP (p=0.043), were significantly changed in the tissue (Bonferoni correction). By using the INTACT database we determined that the pool of all significantly changed proteins interacted with GLUT-4 (27 proteins, p<0.001, Fisher correction) and ACSL-1 (four proteins, p=0.0035, Fisher correction), suggesting that these proteins may be impacted by obesity. The evaluation of the protein expression by means of WB showed that the cardiac expression of ACSL-1 **(D)** (p=0.045) but not GLUT-4 **(E)** was changed (increased) in obese rats. GLUT-1 is the next glucose carrier in cardiomyocytes beyond GLUT-4, and its expression was downregulated in obese rats **(F)** (p=0.026). The Student’s t-test was used for analyzing the data (*p<0.05, ***p<0.001). ACSL-1, long-chain-fatty-acid-CoA ligase 1; AK1, adenylate kinase isoenzyme 1; ATP5L, ATP synthase subunit; GLUT-1, solute carrier family 2, facilitated glucose transporter member 1; GLUT-4, solute carrier family 2, facilitated glucose transporter member 4; HSP70-1, heat shock 70 kDa protein 1A.

By means of global proteomic profiling the pool of 87 proteins significantly changed in the LV of obese rats was identified (**Figure 3**, heatmap), revealing that this phenotype strongly determined the composition of the cardiac tissue (**Figure 4**). Based on the GO database (GO: CC), the analysis of proteomic data revealed that ~36% of significantly different proteins had mitochondrial localization (mitochondrion GO:0005739, p<0.001) and ~27% was cytoplasmic (cytosol GO:0005829, p<0.001) (**Tables S1** and **S2**, Supplemental Information). The data showed that two biological processes (GO: BP) are changed in the hearts of obese rats: the directed movement of phospholipids out of a cell or organelle (phospholipid efflux GO:0033700, p=0.024, **Figure 4**) and the chemical reactions and pathways involving ATP (ATP metabolic process GO:0046034, p=0.043, **Figure 4**). ~47% of the protein pool interacted (INTACT database, Fisher correction) with two proteins: solute carrier family 2, facilitated glucose transporter member 4 (GLUT-4, 27 proteins interacted significantly, p<0.001) and acyl-CoA synthetase long-chain family member 1 (ACSL-1; four proteins interacted significantly, p=0.0035). When the protein pool was analyzed separately depending on whether the proteins were overexpressed or downregulated in the LV of obese rats, the subset of 40 elevated cardiac proteins confirmed the significant interaction with ACSL-1, whereas a subgroup of the remaining proteins (decreased expression in obese animals) interacted with GLUT-4. Thus we decided to perform the western blot to assess the expression of these two molecules. The level of ACSL-1 was significantly increased in the LV of obese rats (**Figure 4**, p=0.045). In spite of the fact that cardiac GLUT-4 is downregulated (29) during insulin resistance, we did not clearly demonstrate its decreased content in the heart of obese rats (**Figure 4**). Rather than by transcriptional mechanisms, the regulation of GLUT-4 depends on its recruitment to the sarcolemma in response to glucose delivery and prolonged hyperinsulinemia leads to the internalization and inactivation of this glucose carrier. In addition to GLUT-4, the most abundant glucose transporter in the heart is solute carrier family 2, facilitated glucose transporter member 1 (GLUT-1) (30), thus it was decided to measure its expression. We have observed the decreased content of GLUT-1 (**Figure 4**, p=0.025), which is an insulin-independent glucose transporter mainly responsible for the basal needs of muscle cells (31). As it was shown that Acsl1 knockout mice upregulate GLUT-1 expression in skeletal (32) and cardiac (33) muscles depending on glucose metabolism, it is possible that that ACSL-1 and GLUT-1 expression depend on each other.

### The Biological Processes Involved in the Regulation of the Immune System and Triglycerides Homeostasis Are Changed in the Plasma of Obese Rats

**Figure 5:**
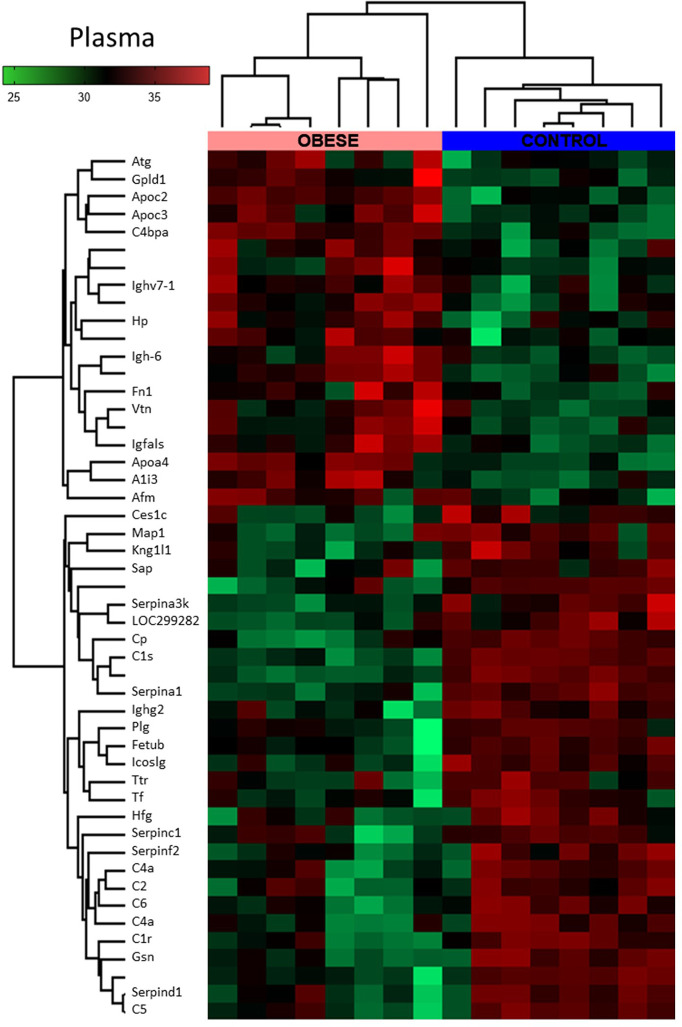
Global proteomic profile of plasma of obese rats. The amount of 49 proteins differed in the plasma of obese rats as compared to homogenous samples from control (n=8). Protein expression data are presented as Z-score transformed.

**Figure 6:**
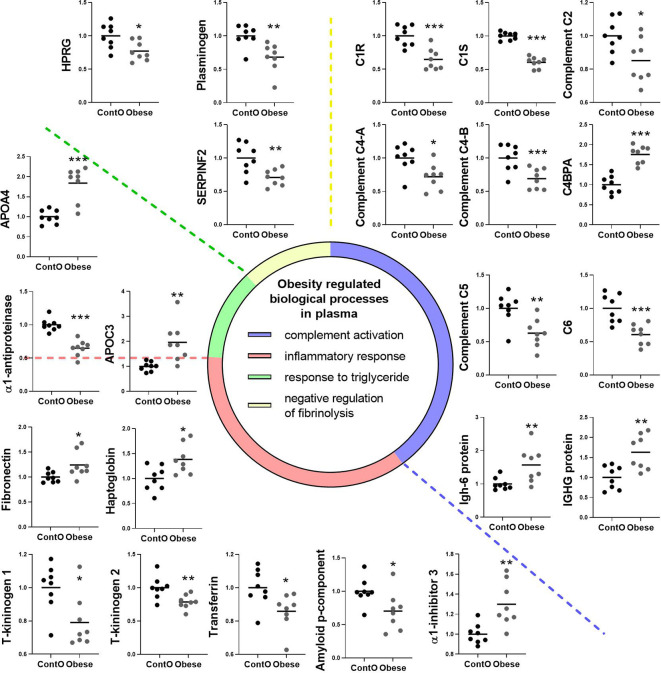
Clustering of the plasma proteins significantly changed in obese rats. Based on the GO : BP (DAVID functional annotation results with Bonferroni statistics) database, plasma proteins were assigned to their respective particular biological function (n=8). Most of the proteins (36%) were involved in the negative regulation of endopeptidase activity (p<0.001, not shown; refer to Supplemental Data for details). 17% of the proteins were involved in immunological regulation (GO:0006956~complement activation, GO:0006958~complement activation). In addition, 17% of molecules participated in the pro-inflammatory response (GO:0006953~acute-phase response, p<0.001; GO:0045087~innate immune response, p<0.01; GO:0006954~inflammatory response, p<0.05). The next biological process changed in the plasma of obese rats included the abnormal level of proteins involved in the regulation of any process that results in a systemic change as a result of a triglyceride stimulus (GO:0034014~response to triglyceride, p<0.05). The pool of proteins (three molecules) involved in processes that stop, prevent, or reduce the frequency, rate, or extent of fibrinolysis resulting in the removal of small blood clots was downregulated in the plasma of obese animals (GO:0051918~negative regulation of fibrinolysis, p<0.05). Data are presented as mean of fold change of control. The Student’s t-test was used for analyzing the data (*p<0.05, **p<0.01, ***p<0.001). APOA4: apolipoprotein A-IV; APOC3: apolipoprotein C-III; C1R: complement C1r subcomponent; C1S: complement C1s subcomponent; C4BPA: C4b-binding protein alpha chain; C6: complement component C6; HPRG: histidine-rich glycoprotein; SERPINF2: serpin family F member 2.

Next, we performed global proteomic measurement in plasma, identifying 41 molecules which differentiate between groups (**Figure 5**, heatmap). Based on the results of DAVID analysis, eight biological processes (GO: BP) were changed in the plasma of obese rats, including i.e. the process that decreases the frequency, rate, or extent of endopeptidase activity (negative regulation of endopeptidase activity GO:0010951, p<0.001), immunological responses (complement activation GO:0006956 and GO:0006958, p<0.001; acute-phase response GO:0006953, p<0.001; inflammatory response GO:0006954, p<0.05), processes that change the state or activity of a cell as a consequence of triglyceride stimulus (response to triglyceride GO:0034014, p<0.05) and process that reduce fibrinolysis (negative regulation of fibrinolysis GO:0051918, p<0.05). **Figure 5** presents the protein involved in these biological processes except for the negative regulation of endopeptidase activity and negative regulation of fibrinolysis (**Tables S3** and **S4**, Supplemental Information). Due to the fact that various molecules participate in different immunologic processes, the proteins of acute and inflammatory responses have been combined (**Figure 6**). Changes of four molecules detected in the LV were also significant in the plasma of obese rats (**Table 1**). Alpha-1-inhibitor III, apolipoprotein A4 (APOA4), and apolipoprotein C3 (APOC3) were increased in both tissues. Transthyretin (TTR; thyroid hormone-binding protein) was elevated in the heart but decreased in the plasma of obese rats.

**Table 1:** List of the proteins significantly changed in both: in the left ventricle of heart and plasma of obese and control (Conto) rats.

Accesion number	Protein name	Obese vs ContO		Function
		Heart	Plasma	
P14046	alpha-1-inhibitor III (A1I3)	↑	↑	Protease inhibitor with a wide spectrum of protein targets.
P02651	apolipoprotein A4 (APOA4)	↑	↑	Major component of HDL and chylomicrons.
A0A0G2K8Q1	apolipoprotein C3 (APOC3)	↑	↑	Inhibits lipoprotein lipase delaying the catabolism of triglyceride-rich particles.
P02767	transthyretin (TTR)	↑	↓	Transport protein that carries the thyroid hormone thyroxine (T4) and retinol-binding protein bound to retinol.

↓ indicates lower protein expression in the obese group when compared to control, while ↑ indicates higher expression in the obese group.

### CR-Induced Weight Loss Partially Restored the Heart’s Architecture, Leaving Fibrotic Debris

**Figure 7:**
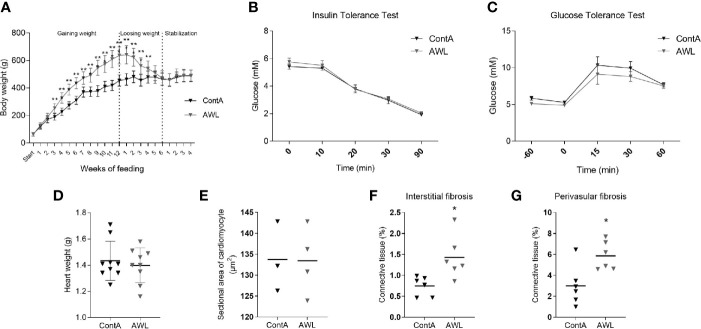
Characteristics of rats after weight loss. After 12 weeks of feeding with a cafeteria diet, rats (n=16) intensively gained weight **(A)**, developing the obesity phenotype. Obese animals were subjected to CR for 6 weeks to lose weight. For the next four weeks, animals from the experimental (After Weight Loss, AWL) and control (ContA) groups received an isocaloric amount of calories (stabilization period) to ensure stable body weight. After weight loss, the rats did not show features of insulin resistance which was confirmed by insulin **(B)** and glucose **(C)** tolerance tests (n=5). The mass of the heart (n=9) **(D)** and size of the cardiomyocytes (n=3-4) **(E)** were restored after weight loss in rats which were previously obese. However, the interstitial **(F)** and perivascular **(G)** amount of connective tissue was elevated in these animals (n=6). Two-way ANOVA with Sidak’s multiple comparison test **(A–C)** and Student’s t-test was used for analyzing the data (*p<0.05, **p<0.01).

Another cohort of young animals was subjected to a cafeteria diet for 12 weeks to gain weight and then to a 6-week-long CR protocol to reduce weight (AWL group; **Figure 7**). The group was accompanied by control animals (ContA group) which were maintained exclusively on a standard diet. This was followed by four subsequent weeks of isocaloric feeding to normalize the systemic parameters between AWL and ContA groups. Obesity phenotype induced insulin insensitivity but applied CR should reverse it, even resulting in higher insulin sensitivity (34). However, there were no significant differences in the response to insulin (**Figure 7**) or glucose (**Figure 7**) in AWL group confirming that insulin sensitivity after the weight loss process was not affected by the bygone obesity and CR. Hyperlipidemia was also reversed after weight loss (**Table S7**, Supplemental Information). We observed that the weight of the collected hearts did not differ among AWL and control animals (**Figure 7**) and the histologic examination did not reveal any differences in the size of the cardiomyocytes among groups (**Figure 7**). However, the amount of interstitial (**Figure 7**) and perivascular (**Figure 7**) connective tissue was elevated in the LV of AWL rats, suggesting persistent cardiac fibrosis in animals which were obese in the past.

### The Identification of Cardiac and Systemic Proteomic Changes Present After Weight Loss

Proteomic data revealed that the level of 14 identified proteins were altered in the hearts of the rats after weight loss (**Table 2**). Two proteins – adenylate kinase 1 (AK1) and complement C3 (C3) – were changed in the hearts of both obese and AWL rats. AK1 was decreased in both obese and AWL groups. However, complement C3 was elevated in the hearts of obese rats but decreased in AWL cohort (**Tables S1** and **S5**, Supplemental Information). The analysis of the plasma revealed that 14 identified proteins varied in the plasma of AWL and control rats (**Table 3**). Antithrombin (p=0.007), complement C5 (p=0.017), heparin cofactor 2 (p<0.001) and transthyretrin (p=0.004; TTR) were noted to be changed in the plasma of obese and AWL rats (**Tables S3** and **S6**, Supplemental Information). These proteins are mostly involved in the regulation of endopeptidase activity (GO:0010951, eight proteins, p<0.001), complement activation (GO:0006957, four proteins, p<0.001), and blood coagulation (GO:0007596, three proteins, p<0.05). The extracellular protein TTR, responsible for the transport of thyroxin and retinol binding protein complex to the various parts of the body, was decreased in the plasma of AWL rats. In the plasma, TTR binds to adipokine known as retinol-binding protein 4 (RBP4), which prevents glomerular filtration and the subsequent catabolism of RBP4 in the kidney. However, despite TTR depletion RBP4 levels were elevated (p=0.048). Several human and animal studies have investigated the influence of high circulating RBP4 levels in the pathogenesis of insulin resistance associated with type 2 diabetes and obesity (35). The current data suggest that the systemic balance between TTR and RBP4 may be disturbed after weight loss, but this phenomenon warrants further evaluation. TTR level was elevated in heart of obese animals but decreased also after weight loss. TTR may be used as a biomarker of stress disorders (36), oxidative stress (37), lean body mass, and catabolic states (38). RBP4 was shown as a marker of insulin resistance (39), but these results are inconclusive (35).

**Table 2:** Proteins significantly changed (Students’s T test, n=8) in the cardiac muscle of rats from AWL (After Weight Loss) group as compared to control (ContA).

UNIPROT	Name: protein (gene)	AWL vs. ContA		Function/biological process
		Fold change	p value	
Q7TMZ9	cardiac titin N2B isoform (N/A)	10.47	0.011	regulates the sarcomere length and muscle function
Q5M819	phosphoserine phosphatase (Psph)	3.73	0.0005	catalyzes the last step in the biosynthesis of serine from carbohydrates
Q5XIG4	OCIA domain containing 1 (Ociad1)	2.07	0.019	regulation of stem cell differentiation
B5DEF6	acyl-CoA dehydrogenase family, member 10 (Acad10)	1.91	0.021	fatty acid beta-oxidation
P51886	lumican (Lum)	1.52	0.014	collagen fibril organization
Q75Q41	translocase of outer mitochondrial membrane 22 (Tomm22)	1.44	0.0064	interacts with TOMM20 and TOMM40, and forms a complex with several other proteins to import cytosolic preproteins into the mitochondrion
P45592	cofilin 1 (Cfl1)	0.78	0.023	controls reversibly actin polymerization and depolymerization in a pH-sensitive manner
P12007	isovaleryl-CoA dehydrogenase (Ivd)	0.76	0.013	mitochondrial matrix enzyme that catalyzes the third step in leucine catabolism
Q6P784	branched chain amino acid transaminase 2 (Bcat2)	0.76	0.049	catalyzes the first reaction in the catabolism of the essential branched chain amino acids leucine, isoleucine, and valine
Q6P9T8	tubulin, beta 4B class IVb (Tubb4b)	0.7	0.035	major constituent of microtubules
**A0A0G2K7Q6**	**adenylate kinase 1 (Ak1)**	**0.67**	**0.019**	catalyzes the reversible transfer of the terminal phosphate group between ATP and AMP. **Downregulated in the heart of obese rats.**
**P01026**	**complement C3 (C3)**	**0.63**	**0.044**	plays a central role in the activation of complement system (a part of the immune system). **Upregulated in the heart of obese rats.**
Q75Q39	translocase of outer mitochondrial membrane 70 (Tomm70)	0.59	0.039	receptor that accelerates the import of all mitochondrial precursor proteins
Q00981	ubiquitin C-terminal hydrolase L1 (Uchl1)	0.12	0.0022	ubiquitin-protein hydrolase involved both in the processing of ubiquitin precursors and of ubiquitinated proteins
				

Values of fold-change <1 indicate lower protein expression in the AWL group when compared to ContA, while values>1 indicate higher expression in the AWL group. Proteins are ranked from largest to smallest fold change. Bolded data – proteins changed in the hearts of both obese and AWL rats.

**Table 3:** Proteins significantly changed (Student’s T test, n=9) in the plasma of rats from AWL (After Weight Loss) group as compared to control individuals (ContA).

UNIPROT	Name: protein (gene)	AWL vs. ContA		Function/biological process
		Fold change	p value	
A0A1K0FUB2	myoglobin (Mb)	31.13	<0.001	serves as a reserve supply of oxygen and facilitates the movement of oxygen within muscles;
E9PSU8	Ig-like domain-containing protein (N/A)	1.94	0.009	involved in a variety of functions, including cell-cell recognition, cell-surface receptors, muscle structure and the immune system;
B2RZC1	retinol binding protein 4 (Rbp4)	1.46	0.047	delivers retinol from the liver stores to the peripheral tissues;
D3ZBS2	inter-alpha trypsin inhibitor, heavy chain 3 (Itih3)	0.9	0.01	a carrier of hyaluronan in serum;
Q5EBC0	inter-alpha-trypsin inhibitor heavy chain family, member 4 (Itih4)	0.9	0.03	type II acute-phase protein (APP) involved in inflammatory responses to trauma;
**Q5M7T5**	**antithrombin (Serpinc1)**	**0.87**	**0.007**	inhibits thrombin as well as other activated serine proteases of the coagulation system, and it regulates the blood coagulation cascade. **Downregulated in the plasma of obese rats.**
M0RBF1	complement C3 (C3)	0.86	0.001	plays a central role in the activation of the complement system;
P20059	hemopexin (Hpx)	0.86	0.01	binds heme and transports it to the liver for breakdown and iron recovery, after which the free hemopexin returns to the circulation;
**A0A096P6L9**	**complement C5 (C5)**	**0.85**	**0.017**	derived from proteolytic degradation of complement C5, C5 anaphylatoxin is a mediator of local inflammatory process. **Downregulated in the plasma of obese rats.**
Q5BKC4	complement C9 (C9)	0.84	0.012	component of the terminal complement complex C5b-9, which induces cleavage and activation of caspase 3 and mediates induction of apoptosis;
Q6MG74	complement factor B (Cfb)	0.8	0.016	complement activation;
P31211	corticosteroid-binding globulin (Serpina6)	0.79	0.005	major transport protein for glucocorticoids and progestins in the blood of almost all vertebrate species;
**A0A0G2K8K3**	**heparin cofactor 2 (Serpind1)**	**0.75**	**<0.001**	thrombin inhibitor activated by the glycosaminoglycans, heparin or dermatan sulfate. **Downregulated in the plasma of obese rats.**
**P02767**	**transthyretin (Ttr)**	**0.65**	**0.004**	thyroid hormone-binding protein. Probably transports thyroxine from the bloodstream to the brain. **Downregulated in the plasma of obese rats.**

Values of fold change <1 indicate lower protein expression in the AWL group when compared to ContA, while values>1 indicate higher expression in the AWL group. Proteins are ranked from largest to smallest fold change. Bolded data – proteins changed in the plasma of both obese and AWL rats.

## Discussion

Here, we discriminate the proteomic and morphological consequences of obesity in the hearts of rats and evaluate whether the changes are reversed by CR-induced weight loss. The altered LV proteomic status and connective tissue build-up associated with weight gain were not completely reversed by body weight normalization.

### Morphological and Molecular Characteristics of the Heart in Obesity

The phenotype of obesity cardiomyopathy includes heart hypertrophy with an increased amount of interstitial and perivascular connective tissue (40). The upregulation of the mTOR pathway is predominantly involved in this cardiac remodeling (41) as a consequence of overfeeding and insulin resistance (42). Therefore, we observed enlarged cardiomyocytes and increased mTOR phosphorylation suggesting the upregulation of this anabolic process in the cardiac cells of obese rats. We assumed that decreased AMPK signaling, as a possible consequence of overfeeding, may be partially involved in the mTOR control, but AMPK phosphorylation and its targeted site on mTOR [Thr2446, leading to its inhibition when phosphorylated (28)] seem to be unaffected, rejecting the assumption above. Further, the observed cardiomyopathy was reflected in a plethora of molecular changes. The proteomic machinery responsible for the transport of triglycerides was boosted in obese animals both in the plasma and the heart muscle. In order to metabolize long-chain fatty acids, they must first be converted to acyl-CoA by ACSL proteins (43). Among the five mammalian ACSL isoforms, ACSL-1 predominates in cardiac tissue (44). The hearts of obese rats expressed more ACSL-1 but had reduced amounts of GLUT-1. These findings suggest that in obesity cardiomyopathy the glucose uptake may be decreased in favor of lipid metabolism. Whereas energetic metabolism in the normal heart depends primarily on ß-oxidation and less on glucose utilization, in the diabetic heart ATP production becomes almost completely reliant on fatty acids oxidation (45, 46), causing the inability to switch between energetic substrates in case of trauma. The inability of efficient ATP generation from glucose under specific pathological conditions contributes to heart failure (47).

Our observations are mostly consistent with results of previous studies applying non-targeted proteomic or transcriptomic methods to screen cardiac tissue in animal models of diet induced obesity. A map of the cardiac proteome in obese rats showed that the proteins involved in regulation of metabolism were predominantly changed (48). The proteomic machinery responsible for fatty acids uptake and oxidation was upregulated in obese animals (48) and expression of the proteins involved in mitochondrial metabolism was affected (49–51). Although the substantial protein expression is changed in obesity the cardiac proteome may be also affected in a different way, by modifying pattern of acetylation (52). Obesity also results in cardiac transcriptome modifications that can be associated with a number of pathologies including the cardiac hypertrophy. Changes of gene expression that occur during hypertrophic cardiac remodeling arise as a consequence of mechanical overloading and play a critical role in normal cardiac function and pathogenesis of heart [well discussed in (53)]. Studies of global gene profiling in rodents have identified differentially expressed transcripts in the cardiac ventricle. The expression of genes of lipid and protein metabolism, fatty acid beta-oxidation, cell death, apoptosis, peroxisome organization, and biogenesis were upregulated in hearts of obese db/db mice (54). A more recent multi-omics analysis revealed that cardiac lipid metabolism was changed in type-2 diabetic db/db mice which was supported by metabolomics and transcriptomics (55). In mice fed, an obesogenic diet global gene expression analysis revealed obesity related changes in glucose metabolism pathways (56). In line with such transcriptomic data, the proteomic results presented here confirm that obese phenotype propagates cardiac lipid utilization whilst simultaneously reducing glucose metabolism homeostasis. Numerous reports have demonstrated increased myocardial fatty acid utilization in obese animal models and cardiac lipotoxicity has been recognized as an important contributor to cardiovascular complications of obesity (57). Costa and Franco provided a broad view on cardiac transcriptome changes produced by obesity showing that expression of genes involved in cellular architecture and lipid metabolism was affected which supports our data (53). The regulation of gene expression may be a consequence of the plethora of epigenetic modification present in cardiometabolic diseases and epigenetic therapies may represent a new frontier in cardiovascular medicine (58).

The proteomic composition of the plasma in obese rats strongly supports the notion that the systemic consequences of obesity and the cafeteria diet are reflected in the cardiac muscle. Increased angiotensinogen levels led to the suspicion that obese rats developed hypertension with the involvement of the renin–angiotensin system (59), and this could contribute to the overgrowth of cardiomyocytes (60). APOA4 and APOC3 apolipoproteins were increased in the plasma of obese rats and four apolipoproteins were elevated in the heart (A1, APOA4, APOC3, and E), suggesting the high vulnerability of cardiac tissue toward the lipidomic changes accompanying diet-induced obesity. Decreased levels of gelsoline indicates pathological conditions as this protein normally occurs in high amounts and is extensively degraded when binding to filamentous actin (released upon cell death or rupture) occurs (61). This protein mitigates the detrimental effects of systemic inflammation (62), thus gelsoline downregulation could potentiate a greater impact of obesity-related inflammation on the system. Protein clustering revealed that obese animals developed contradictory inflammatory status. On the one hand, chronic inflammation elevated pro-inflammatory molecules such as fibronectin (63) and haptoglobin (64) in obese animals. Inconsistently, α1-inhibitor-3 (65) and kininogens (66) known as pro-inflammatory factors were decreased. This was associated with the downregulation of anti-inflammatory α1-antitrypsin (67) and transferrin, which is negatively regulated during the acute phase (68). On the other hand, the defense response could be diminished, which was recognized as the decreased expression of the complement system proteins being a part of the immune system launched during a pathogen attack (69).

### Weight Loss Reverse Cardiac Overgrowth But Not Fibrotic Deposition

Obesity evokes cardiac remodeling, which seems to be rescued after weight loss (18, 40). However, a significant finding of our study was that after weight loss the heart is still abundantly composed of connective tissue. We suspect that this could be a residue of obesity as well as a consequence of CR applied for weight reduction. Whereas intracellular compartments in the cardiomyocytes are degraded *via* autophagy, allowing for tissue rearrangement, the redundant ECM is proteolyzed and then digested by the phagocytic cells of the complement immune system (70). The proteins of the complement cascade (C3, C5, C9, and factor B) were downregulated after weight loss, suggesting that systemic inflammatory response and the recruitment of phagocytic cells was rather repressed than mobilized possibly diminishing the complete restoration of tissues during weight loss. Due to the novelty of this thesis, more research is required to support its authenticity. However, there may be an additional reason for the observed elevation of the heart’s connective tissue after weight reduction. CR impacts cardiac morphology in an age-dependent manner by stimulating the fibrotic deposition in young-adult mice (age-matched to our rats), but not in older animals, emphasizing the possible fibrotic residue of CR in the heart (71).

The different proteomic composition of the LV in AWL rats may be a consequence of cardiac fibrosis and ECM excess, which is illustrated by the lower amount of cardiac tubulin as this protein is abundantly expressed in the myocytes but scarce in ECM (72). Conversely, the pool of lumican, the main components of cardiac ECM was elevated in the hearts of AWL rats (73). Lumican expression is increased in hepatic (74), pulmonary (75), and cardiac (73) fibrosis. On the one hand, experimental and clinical findings revealed that the overexpression of lumican in cardiac fibroblasts is evoked during heart failure (73). This protein regulates cardiac remodeling following LV pressure overload (76) and has been shown to have a role in ECM remodeling and fibrosis in different cardiovascular diseases (77–79). On the other hand, lumican is required for cardiac remodeling by ensuring the structural integrity of connective tissue and survival following pressure overload (76). Possibly lumican may play a role in obesity-related cardiac remodeling and we observed that its level is elevated after weight loss, carrying the possible risk of fibrosis-related disturbances.

We examined dietary modification as a factor for weight loss but it will be valuable to extend this investigation in the future by adding physical activity as an important element of obesity treatment. Weight loss through diet and exercise appears an effective therapy to reduce cardiovascular risk associated with obesity (80, 81); rodent studies indicate that both the cardioprotective (82, 83) and gene expression (84) effects of exercise are proportional to its intensity. Also in the skeletal muscle the administration of high fat diet enhanced lipid catabolism at the transcriptional level which was probably a compensatory mechanism in response to lipid overload but after acute exercise the pattern of gene expression changed dramatically (85). The beneficial remodeling and metabolic effects of exercise training in cardiac and skeletal muscle of obese mice depend on autophagy (86). Further research is needed to validate the long term outcomes of dietary and/or exercise induced weight loss on restoration of homeostasis affected by obesity.

### Possible Biomarkers to Predict Cardiac Recovery After Weight Loss

We measured proteins in the heart and plasma in obese rats and animals subjected to weight loss. This approach gave us an opportunity to propose tissue specific and blood protein signatures of obesity cardiomyopathy as predictors of disease and response to therapy. Particularly we propose that markers of lipid storage and metabolism may warrant consideration for monitoring therapeutic progress of weight loss because cardiac lipotoxicity is associated with structural remodeling and functional changes that are the features of obesity-related cardiomyopathy (87). APOC3 and APOA4 are two potential protein candidates of obesity cardiomyopathy signatures which levels were elevated in cardiac muscle and plasma but rescued after weight loss. Increased levels of both proteins are associated with the risk of cardiovascular events (88, 89). The critical role of lipotoxicity in cardiomyopathy is supported by the observation that ACSL-1 was upregulated in the heart of obese animals suggesting propagation of cardiac lipid accumulation. Acsl-1 gene modulation may be a potential therapeutic strategy in obesity-related cardiomyopathy and Acsl-1 haploinsufficiency resulted in normalization of cardiac lipid storage in db/db mice (90). ACSL-1 level can be monitored in peripheral blood leukocytes and it was shown that it may be a molecular marker when determining the risk of acute myocardial infarction in humans (91). It may be also monitored by assessing its epigenetic marks because Acsl-1 is regulated by methylation, and hypometylation was observed in obese humans (whole-blood DNA) (92) and mice (adipose tissue) (93). Future research could consider a pharmacological obesity co-treatment by triacsin C with evaluation of its cardioprotective effects because this fungal metabolite has been recognized as ACSL-1 inhibitor (94). ACSL-1 may be regulated by dietary fat consumption (95) raising the importance of adequate nutritional strategy in obesity treatment. Cardiac level of C3 was elevated in obese rats but downregulated after weight loss. Inflammation is involved in cardiac remodeling and activation of complement cascade accompanies cardiovascular disturbances both in humans and rodents (96, 97). C3 level has been shown to be elevated in serum of patients with left ventricular hypertrophy (98), in hypertensive patients (99) and more recently was recognized as a marker of hypertrophic cardiomyopathy (100). However we could not point at the immune proteins as biomarkers for monitoring the cardiac recovery because we observed upregulation of complement cascade proteins in cardiac muscle but not in the plasma. Intriguingly we found that the level of cardiac AK1 - downregulated in obese animals - was still low despite the weight normalization. This enzyme catalyze the nucleotide phosphoryl exchange reaction 2ADP ↔ ATP + AMP being a critical player in metabolic monitoring and systemic integration of different signaling pathways. The protein plays an important role in cardiomyocytes and the evidence is mounting regarding the direct relationship between defects in AK1 and AMP metabolic signaling in human diseases, such as heart failure, hypertrophic cardiomyopathy, diabetes and obesity (101). Reduction in total AK1 protein expression was observed in failing hearts, whereas AK1 mRNA levels and enzyme activity remained unchanged (102). Persistent downregulation of AK1 may point to increased cardiac risk despite weight loss, emphasizing the need for recognizing the cardiac outcomes of previous obesity.

### Limitations of the Study and Transferal of the Results

In the study we have shown proteomic and structural changes of the heart in obese rats and after weight loss. The rat model of diet-induced obesity was introduced to reflect developmental obesity. Thus the animals were young (postnatal day 28) at the time the obesogenic diet was introduced, reflecting ~8 months of human age (103) when infants begin receiving solid food (104). The overconsumption of protein and sugars especially induce developmental obesity (105), and the global intake of the latter range from 1.9% to 13.4% in humans before 2 years of age (106) underlies the high dietary risk factors for the development of obesity in infants. Comprehensive proteomic evaluation was performed in the plasma and cardiac tissue of obese and CR-cured rats (with adequate, age-matched controls). In the second group, the tissue material was collected after four weeks of isocaloric intake, where AWL rats and control companions received the same amount of calories, allowing for weight maintenance without significant weight gain or reduction. This approach seemed to be the most reasonable and made it somewhat possible to eliminate the systemic effects of CR (107). The animals could not be fed by standard chow in an ad libitum manner because from our experience, AWL rats regain weight more extensively than control. Thus, during the “stabilization state” both groups received ~100% of calorie needs. We did not find any justification to compare the effects observed in obese animals with AWL rats and adequate controls (by comparing the four groups in parallel), especially considering the different age of the animals. In such a comparison, we would not estimate the impact of body weight only, but also the age factor, thereby falsifying the conclusion. A limitation of the current study is the lack of functional data that directly corresponds with our structural and proteomic results. However it is well established that rats fed with cafeteria diet develop functional consequences such as elevated blood pressure, high heart rate, affected hyperpolarization and autonomic dysfunctions (108). Moreover, robust alteration in the myocardial proteome of diet-induced obese rats can be detected even before the severe functional impairment occurs (48). We believe that our model reflects well the cardiac perturbations of the heart in obesity. Future work should demonstrate areas of overlap between proteomic correlates and functional activation correlates in the same experimental design.

The prevalence of obesity actually increased with hordes of young people predisposed to weight-loss therapies. If adequate approaches will be applied to reduce body weight, these patients could be rescued from obesity within a few years (109). The restoration of a lean phenotype masks the obesity experience, possibly shaping future diagnosis in adulthood or the elderly. However, the persistent effects of obesity are not well explored. We suggest that clinicians consider the possibility of persisting cardiac consequences of prior obesity in lean patients during diagnosis.

## Conclusion

Global proteomic profiling with morphological evaluation was performed in the hearts of obese rats and after weight loss. We conclude that obesity cardiomyopathy is highly complex, integrating anabolic, metabolic, and immunogenic complications in the cells. The systemic status of overfeeding, hyperlipidemia, and insulin resistance may contribute to the cardiac adjustment developed during weight gain. After losing weight, the heart’s phenotype can be ostensibly restored to normal. However, some abnormalities still occur at the morphological (fibrosis) and proteomic levels.

## Data Availability Statement

The mass spectrometry proteomics data have been deposited to the ProteomeXchange Consortium via the PRIDE partner repository with the dataset identifier PXD019461 (http://www.ebi.ac.uk/pride).

## Ethics Statement

The animal study was reviewed and approved by Local Ethics Committee for Animal Experimentation in Katowice.

## Author Contributions

AL: conceptualization, methodology, investigation, writing - original draft, funding acquisition. ŁM: investigation, formal analysis. KB: investigation. DL: investigation, writing - review and editing. MP: investigation. JL-K: supervision. All authors reviewed the manuscript. All authors contributed to the article and approved the submitted version. This manuscript has been released as a pre-print at Research Square (110).

## Funding

This work was primarily supported by the Nutricia Foundation [grant number RG 1/2017] and partially by statutory grants: KNW-1-173/K/9/0, KNW-1-153/K/9/0 from the Medical University of Silesia, Katowice, Poland and AWF/NF/2019/1 from the Jerzy Kukuczka Academy of Physical Education, Katowice, Poland.

## Conflict of Interest

The authors declare that the research was conducted in the absence of any commercial or financial relationships that could be construed as a potential conflict of interest.
