# Supplementary material for: Proteomic and Structural Manifestations of Cardiomyopathy in Rat Models of Obesity and Weight Loss
Source: Front Endocrinol (Lausanne). 2021 Feb 24;12:568197. doi: 10.3389/fendo.2021.568197 (PMC7945951; doi:10.3389/fendo.2021.568197)
Supplement: Supplementary file 1 [file DataSheet_1.docx]

***Supplementary Material***

**Supplementary Tables (S1-7)**

**Supplementary Figures (S1-33)**

The mass spectrometry proteomics data have been deposited to the ProteomeXchange Consortium via the PRIDE* partner repository with the dataset identifier PXD019461 (<http://www.ebi.ac.uk/pride>).

*Perez-Riverol Y, Csordas A, Bai J, Bernal-Llinares M, Hewapathirana S, Kundu DJ, Inuganti A, Griss J, Mayer G, Eisenacher M, et al. The PRIDE database and related tools and resources in 2019: improving support for quantification data. Nucleic Acids Res (2019) 47:D442–D450. doi:10.1093/nar/gky1106

# **Supplementary Tables (S1-8):**

**Table S1** List of significantly changed proteins in the left ventricles of hearts from control and obese rats identified by LC-MS/MS (Student’s T test) (n=5).

| Accesion number | Protein name | Gene name | Peptide count (unique peptides) | Confidence score | Sequence coverage (%) | p-value | Fold change* |
| --- | --- | --- | --- | --- | --- | --- | --- |
| A0A1W2Q6E9 | Moesin | Msn | 11 (6) | 64.579 | 25 | 0.020 | -0.3 |
| A0A0G2JSU4 | Protein NDRG2 | Ndrg2 | 12 (12) | 323.31 | 63.9 | 0.011 | -0.3 |
| A0A0G2JUA5 | AHNAK nucleoprotein | Ahnak | 76 (1) | 323.31 | 43.3 | 0.029 | -0.4 |
| A0A0G2JV81 | ADP-ribosylation factor 1 | Arf1 | 6 (6) | 21.016 | 43.6 | 0.007 | 0.3 |
| A0A0G2JVL6 | NADH dehydrogenase [ubiquinone] 1 alpha subcomplex subunit 8 | Ndufa8 | 9 (9) | 198.16 | 61 | 0.043 | 0.2 |
| Q6IMY8 | Heterogeneous nuclear ribonucleoprotein U, hnRNP U | Hnrnpu | 4 (4) | 31.869 | 8.4 | 0.004 | -0.3 |
| A0A0G2JZA2 | GrpE protein homolog 1, mitochondrial | Grpel1 | 5 (5) | 35.482 | 28.7 | 0.040 | 0.4 |
| A0A0G2K135 | Complement factor I | Cfi | 6 (6) | 27.825 | 14.7 | 0.027 | 0.9 |
| Q65ZS7 | Apolipoprotein E | Apoe | 9 (9) | 155.75 | 34.5 | 0.045 | 0.6 |
| A0A0G2K4U2 | Glutathione S-transferase, mu 7 | Gstm7 | 10 (0) | 39.839 | 53.2 | 0.034 | -0.6 |
| A0A0G2K890 | Ezrin | Ezr | 7 (3) | 22.206 | 13.5 | 0.033 | -0.4 |
| A0A0G2K8Q1 | Apolipoprotein C-III | Apoc3 | 2 (2) | 89.746 | 39 | 0.023 | 1.2 |
| A0A0H2UHM5 | Protein disulfide-isomerase A3 | Pdia3 | 15 (15) | 62.677 | 35.3 | 0.008 | 0.5 |
| A2VD12 | Pre-B-cell leukemia transcription factor-interacting protein 1 | Pbxip1 | 11 (11) | 58.301 | 24 | 0.023 | 0.8 |
| M0RDM4 | Histone H2A | LOC680322 | 4 (1) | 183.87 | 50 | 0.045 | 0.7 |
| D3ZK97 | Histone H3 | H3f3c | 3 (1) | 90.26 | 35.3 | 0.025 | -1.9 |
| B0BNM1 | NAD(P)H-hydrate epimerase | Apoa1bp | 7 (7) | 36.514 | 46.8 | 0.005 | -0.7 |
| B2GV06 | Succinyl-CoA:3-ketoacid coenzyme A transferase 1, mitochondrial | Oxct1 | 17 (17) | 323.31 | 54.8 | 0.002 | -0.8 |
| B2RYS8 | NADH dehydrogenase [ubiquinone] 1 beta subcomplex subunit 8, mitochondrial | Ndufb8 | 8 (8) | 83.499 | 57.5 | 0.014 | 0.6 |
| B4F789 | Apolipoprotein B editing complex 2 (Predicted), isoform CRA_a | Apobec2 | 9 (9) | 118.88 | 67.4 | 0.001 | -0.7 |
| B6DYQ2 | Glutathione S-transferase Mu 2 | Gstm2 | 14 (7) | 120.99 | 64.7 | <0.001 | -0.7 |
| B6DYQ7 | Glutathione S-transferase P | Gstp1 | 5 (5) | 59.368 | 32.4 | 0.043 | -1.4 |
| C0JPT7 | Filamin A (Filamin alpha) | Flna | 15 (12) | 56.676 | 8.9 | 0.019 | 0.6 |
| O35802 | Inter alpha-trypsin inhibitor, heavy chain 4 | ITIH4 | 18 (18) | 164.28 | 30.5 | 0.027 | 0.9 |
| D3ZG43 | NADH dehydrogenase [ubiquinone] 1 alpha subcomplex assembly factor 3 | Ndufs3 | 13 (13) | 267.08 | 66.3 | 0.021 | -0.3 |
| D3ZH76 | PPARGC1 and ESRR-induced regulator, muscle 1 | Perm1 | 4 (4) | 79.704 | 12.9 | 0.002 | 1.8 |
| D3ZQN7 | Laminin subunit beta 1 | Lamb1 | 18 (18) | 178.45 | 14.8 | 0.024 | -0.3 |
| D3ZVB7 | Osteoglycin | Ogn | 8 (8) | 116.35 | 34.2 | 0.023 | -0.7 |
| D3ZW08 | Adenylosuccinate lyase | Adsl | 5 (5) | 32.497 | 20.7 | 0.041 | 0.7 |
| D4A565 | NADH dehydrogenase (Ubiquinone) 1 beta subcomplex | Ndufb5 | 5 (5) | 20.555 | 23.8 | 0.037 | 0.5 |
| D4A6E3 | Murinoglobulin-1 | Mug1 | 54 (0) | 323.31 | 51.6 | 0.028 | 0.7 |
| F1LN88 | Aldehyde dehydrogenase, mitochondrial | Aldh2 | 22 (22) | 323.31 | 67.4 | 0.034 | -0.2 |
| F1LRJ9 | Methanethiol oxidase | Selenbp1 | 22 (22) | 255.48 | 62.1 | 0.005 | -0.5 |
| F1LWG8 | Sarcalumenin | Srl | 25 (25) | 323.31 | 35.1 | 0.014 | -0.3 |
| F1M820 | Sorbin and SH3 domain-containing protein 1 | Sorbs1 | 19 (0) | 31.151 | 21.5 | 0.012 | 1.2 |
| F1M944 | Calsequestrin | Casq2 | 15 (15) | 323.31 | 49.6 | 0.028 | -0.3 |
| F2Z3Q8 | Importin subunit beta-1 | Kpnb1 | 6 (6) | 19.104 | 12.9 | 0.029 | 0.8 |
| G3V7J0 | Methylmalonate-semialdehyde dehydrogenase [acylating], mitochondrial | Aldh6a1 | 22 (22) | 323.31 | 56.1 | 0.009 | -0.2 |
| Q2XTA9 | Adrenodoxin, mitochondrial | Fdx1 | 2 (2) | 30.735 | 39 | 0.049 | 0.9 |
| G3V885 | Myosin-6 | Myh6 | 152 (1) | 323.31 | 66.1 | 0.043 | -0.2 |
| G3V8B0 | Myosin-7 | Myh7 | 141 (37) | 323.31 | 62.6 | 0.013 | -1.1 |
| M0R7S5 | Perilipin 4 | Plin4 | 7 (7) | 19.455 | 27.1 | 0.045 | -1.6 |
| M0RAK2 | RCG22622 | LOC684270 | 3 (3) | 105.6 | 28.7 | 0.026 | -1.6 |
| M0RBF1 | Complement C3 | C3 | 66 (58) | 323.31 | 57.1 | 0.012 | 0.5 |
| O35244 | Peroxiredoxin-6 | Prdx6 | 14 (14) | 323.31 | 72.3 | 0.047 | -0.4 |
| P00507 | Aspartate aminotransferase, mitochondrial | Got2 | 23 (23) | 323.31 | 63.3 | 0.020 | -0.4 |
| P02651 | Apolipoprotein A-IV | Apoa4 | 17 (17) | 323.31 | 62.9 | 0.001 | 1.0 |
| P02680 | Fibrinogen gamma chain | Fgg | 12 (12) | 208.82 | 36 | 0.012 | 0.6 |
| P02767 | Transthyretin | Ttr | 5 (5) | 122.75 | 59.9 | 0.016 | 1.0 |
| P02793 | Ferritin light chain 1 | Ftl1 | 6 (6) | 122.08 | 53 | 0.005 | -1.1 |
| P04639 | Apolipoprotein A-I | Apoa1 | 14 (14) | 274.05 | 57.1 | 0.027 | 0.8 |
| P04797 | Glyceraldehyde-3-phosphate dehydrogenase | Gapdh | 18 (7) | 323.31 | 71.8 | 0.040 | -0.3 |
| P0DMW1 | Heat shock 70 kDa protein 1B | Hspa1a | 10 (5) | 34.747 | 27.3 | 0.042 | -0.5 |
| P10888 | Cytochrome c oxidase subunit 4 isoform 1, mitochondrial | Cox4i1 | 11 (11) | 43.468 | 65.7 | 0.010 | 0.6 |
| P13803 | Electron transfer flavoprotein subunit alpha, mitochondrial | Etfa | 14 (14) | 323.31 | 63.1 | 0.013 | -0.3 |
| P14046 | Alpha-1-inhibitor 3 | A1i3 | 55 (15) | 323.31 | 52.8 | 0.001 | 0.9 |
| P14408 | Fumarate hydratase, mitochondrial | Fh | 15 (15) | 323.31 | 49.1 | 0.015 | -0.3 |
| P14669 | Annexin A3 | Anxa3 | 8 (8) | 26.593 | 32.4 | 0.026 | -0.6 |
| P16290 | Phosphoglycerate mutase 2 | Pgam2 | 15 (13) | 276.46 | 65.2 | 0.048 | 0.3 |
| P23928 | Alpha-crystallin B chain | Cryab | 10 (10) | 323.31 | 70.9 | 0.043 | -0.5 |
| P27605 | Hypoxanthine-guanine phosphoribosyltransferase | Hprt1 | 10 (10) | 78.991 | 65.6 | 0.047 | -0.7 |
| P35213 | 14-3-3 protein beta/alpha | Ywhab | 7 (5) | 80.02 | 35 | 0.040 | -0.3 |
| P39069 | Adenylate kinase isoenzyme 1 | Ak1 | 9 (9) | 288.86 | 49 | 0.013 | -0.9 |
| P41562 | Isocitrate dehydrogenase [NADP] cytoplasmic | Idh1 | 11 (10) | 67.315 | 34.1 | 0.021 | -1.0 |
| P47967 | Galectin-5 | Lgals5 | 5 (5) | 32.664 | 43.4 | 0.003 | 0.9 |
| P52944 | PDZ and LIM domain protein 1 | Pdlim1 | 10 (10) | 132.17 | 54.7 | 0.042 | -0.6 |
| P62632 | Elongation factor 1-alpha 2 | Eef1a2 | 14 (9) | 323.31 | 51 | 0.018 | -0.4 |
| P62870 | Transcription elongation factor B polypeptide 2 | Tceb2 | 5 (5) | 15.057 | 38.1 | 0.009 | 0.6 |
| P63331 | Serine/threonine-protein phosphatase 2A catalytic subunit alpha isoform | Ppp2ca | 7 (7) | 87.228 | 40.5 | 0.043 | -0.9 |
| P85834 | Elongation factor Tu, mitochondrial | Tufm | 21 (21) | 323.31 | 59.5 | 0.018 | 0.2 |
| P97584 | Prostaglandin reductase 1 | Ptgr1 | 6 (6) | 75.329 | 27.7 | 0.047 | -0.8 |
| Q03626 | Murinoglobulin-1 | Mug1 | 54 (0) | 62.978 | 52.2 | 0.001 | 0.7 |
| Q4V8H8 | EH domain-containing protein 2 | Ehd2 | 20 (19) | 284.16 | 51.7 | 0.031 | -1.0 |
| Q5PQK2 | FUS RNA-binding protein | Fus | 3 (3) | 15.195 | 9.7 | 0.005 | 1.7 |
| Q5XIG1 | Ldb3 protein | Ldb3 | 15 (1) | 11.869 | 80.2 | 0.044 | -0.8 |
| Q5XIN6 | LETM1 and EF-hand domain-containing protein 1, mitochondrial | Letm1 | 10 (10) | 107.96 | 24.4 | 0.039 | 0.5 |
| Q62651 | Delta(3,5)-Delta(2,4)-dienoyl-CoA isomerase, mitochondrial | Ech1 | 12 (12) | 323.31 | 48.3 | 0.049 | 0.4 |
| Q63041 | Alpha-1-macroglobulin | A1m | 58 (58) | 323.31 | 59.8 | 0.033 | 0.6 |
| Q63080 | Ca2+/Mg2+ ATPase | N/A | 16 (1) | 3.2713 | 33.7 | 0.036 | -1.7 |
| Q63362 | NADH dehydrogenase [ubiquinone] 1 alpha subcomplex subunit 5 | Ndufa5 | 9 (9) | 238.4 | 68.1 | 0.031 | -0.7 |
| Q66HF3 | Electron transfer flavoprotein-ubiquinone oxidoreductase, mitochondrial | Etfdh | 20 (20) | 302.72 | 50.3 | 0.014 | 0.5 |
| Q68FY4 | Vitamin D-binding protein | Gc | 20 (20) | 323.31 | 67.4 | 0.048 | 0.6 |
| Q6GMN8 | Alpha-actinin-1 | Actn1 | 24 (4) | 56.257 | 26.8 | 0.032 | -1.1 |
| Q6P0K8 | Junction plakoglobin | Jup | 19 (19) | 194.55 | 40.7 | 0.042 | -0.6 |
| Q6PDU7 | ATP synthase subunit g, mitochondrial | Atp5l | 8 (8) | 142.63 | 82.5 | 0.042 | 0.8 |
| Q7M0E3 | Destrin | Dstn | 6 (6) | 24.158 | 46.1 | 0.011 | 0.6 |
| Q9JJW3 | Up-regulated during skeletal muscle growth protein 5 | Usmg5 | 4 (4) | 49.429 | 84.5 | 0.044 | 0.4 |

*Values of negative fold-change indicate lower protein expression in the obese group when compared to control, while positive values indicate higher expression in the obese group.

**Table S2** Gene ontology (GO)-term enrichment analysis performed with the DAVID Bioinformatics Resources 6.8 from the pool of the proteins significantly changed in the left ventricles of hearts from control and obese rats.

| **Category** | **Term** | **Count** | **%** | **Fold Enrichment** | **Bonferroni p value** |
| --- | --- | --- | --- | --- | --- |
| GOTERM_CC_DIRECT | GO:0005739~mitochondrion | 31 | 36.05 | 4.32 | 5.66E-10 |
| GOTERM_CC_DIRECT | GO:0072562~blood microparticle | 10 | 11.63 | 18.91 | 5.52E-07 |
| GOTERM_CC_DIRECT | GO:0005615~extracellular space | 22 | 25.58 | 3.73 | 3.96E-05 |
| GOTERM_CC_DIRECT | GO:0005829~cytosol | 23 | 26.74 | 3.23 | 2.17E-04 |
| GOTERM_CC_DIRECT | GO:0005743~mitochondrial inner membrane | 11 | 12.79 | 7.84 | 2.63E-04 |
| GOTERM_CC_DIRECT | GO:0043209~myelin sheath | 9 | 10.47 | 10.51 | 4.49E-04 |
| GOTERM_CC_DIRECT | GO:0005925~focal adhesion | 11 | 12.79 | 6.20 | 0.002 |
| GOTERM_CC_DIRECT | GO:0030018~Z disc | 7 | 8.14 | 13.35 | 0.002 |
| GOTERM_CC_DIRECT | GO:0042627~chylomicron | 4 | 4.65 | 81.14 | 0.002 |
| GOTERM_CC_DIRECT | GO:0005913~cell-cell adherens junction | 9 | 10.47 | 7.75 | 0.004 |
| GOTERM_CC_DIRECT | GO:0048471~perinuclear region of cytoplasm | 13 | 15.12 | 4.52 | 0.01 |
| GOTERM_BP_DIRECT | GO:0033700~phospholipid efflux | 4 | 4.65 | 61.10 | 0.02 |
| GOTERM_CC_DIRECT | GO:0034361~very-low-density lipoprotein particle | 4 | 4.65 | 52.50 | 0.01 |
| GOTERM_BP_DIRECT | GO:0046034~ATP metabolic process | 5 | 5.81 | 23.24 | 0.04 |
| GOTERM_CC_DIRECT | GO:0005747~mitochondrial respiratory chain complex I | 5 | 5.81 | 19.92 | 0.02 |
| GOTERM_CC_DIRECT | GO:0005737~cytoplasm | 40 | 46.51 | 1.70 | 0.03 |

**Table S3** List of significantly changed proteins in the plasma collected from control and obese rats identified by LC-MS/MS (Student’s T test) (n=9).

| Accession number | Protein name | Gene name | Peptide count (unique peptides) | Confidence score | Sequence coverage (%) | p-value | Fold change* |
| --- | --- | --- | --- | --- | --- | --- | --- |
| A0A096P6L9 | Complement C5 | C5 | 53 (50) | 323.31 | 51.2 | 0.010 | -0.71239 |
| A0A0G2JY31 | Alpha-1-antiproteinase | Serpina1 | 28 (28) | 323.31 | 64 | <0.001 | -0.64127 |
| A0A0G2JYK0 | Serine protease inhibitor A3N | LOC299282 | 31 (0) | 323.31 | 48.8 | 0.001 | -0.41918 |
| A0A0G2JZS9 | Ig-like domain-containing protein | N/A | 3 (3) | 33.621 | 42.3 | 0.016 | 0.829278 |
| A0A0G2K3A6 | Ig-like domain-containing protein | N/A | 4 (0) | 79.935 | 58.2 | 0.004 | 0.714521 |
| A0A0G2K9Y5 | Histidine-rich glycoprotein | Hrg | 20 (20) | 323.31 | 48.3 | 0.017 | -0.37038 |
| A0A0G2K4K2 | Ig-like domain-containing protein | N/A | 3 (2) | 55.891 | 41.7 | 0.030 | 0.467175 |
| A0A0G2K7X7 | Complement C7 | C7 | 18 (18) | 168.09 | 30 | 0.001 | -1.00738 |
| A0A0G2K828 | Ig-like domain-containing protein | N/A | 5 (4) | 98.135 | 58.8 | <0.001 | -4.15247 |
| A0A0G2K8K3 | Heparin cofactor 2 | Serpind1 | 18 (18) | 323.31 | 48.5 | 0.012 | -0.62075 |
| A0A0G2K8Q1 | Apolipoprotein C-III | Apoc3 | 4 (4) | 323.31 | 51 | 0.001 | 0.898349 |
| G3V7K3 | Ceruloplasmin | Cp | 63 (63) | 323.31 | 68 | <0.001 | -0.42401 |
| H6X338 | Serum amyloid P-component | sap | 7 (7) | 238.74 | 50.4 | 0.026 | -0.58371 |
| A0A0H2UHM3 | Haptoglobin | Hp | 30 (30) | 323.31 | 65.9 | 0.014 | 0.481676 |
| B5DEH7 | C1r protein | C1r | 16 (1) | 286.17 | 38.8 | <0.001 | -0.65069 |
| D4ACR1 | Ig-like domain-containing protein | Ighv7-1 | 6 (5) | 58.613 | 68.6 | 0.001 | -1.58023 |
| F1LRE2 | Insulin-like growth factor binding protein, acid labile subunit, isoform CRA_b | Igfals | 20 (1) | 323.31 | 45.6 | 0.002 | 0.318309 |
| F1LST1 | Fibronectin | Fn1 | 85 (85) | 323.31 | 56.1 | 0.022 | 0.294522 |
| F1LVL2 | Inducible T-cell co-stimulator ligand | Icoslg | 3 (3) | 77.535 | 10.7 | 0.002 | -0.73575 |
| F1LXY6 | Ig-like domain-containing protein 7-1 | Ighv7-1 | 7 (7) | 204 | 80 | 0.008 | 0.622298 |
| F1M663 | Ig-like domain-containing protein | N/A | 2 (1) | 42.965 | 28.7 | 0.008 | 0.924509 |
| Q68FT8 | RCG33981, isoform CRA_a | Serpinf2 | 15 (15) | 323.31 | 47.3 | 0.007 | -0.48155 |
| G3V7L3 | Complement C1s subcomponent | C1s | 14 (1) | 323.31 | 34.1 | <0.001 | -0.73246 |
| G3V8B1 | Phosphatidylinositol-glycan-specific phospholipase D | Gpld1 | 28 (28) | 323.31 | 53.4 | 0.008 | 0.353136 |
| G3V8D4 | Apolipoprotein C-II | Apoc2 | 5 (5) | 170.79 | 49.5 | <0.001 | 0.862446 |
| M0R692 | Ig-like domain-containing protein | N/A | 5 (2) | 81.733 | 73.5 | 0.011 | 1.041664 |
| M0R9U2 | Ig-like domain-containing protein | N/A | 8 (8) | 255.3 | 85.7 | <0.001 | 2.09089 |
| P01015 | Angiotensinogen | Agt | 17 (17) | 323.31 | 46.8 | 0.021 | 0.270329 |
| P01048 | T-kininogen 1 | Map1 | 25 (0) | 323.31 | 52.3 | 0.012 | -0.34628 |
| P02651 | Apolipoprotein A-IV | Apoa4 | 50 (50) | 323.31 | 88 | <0.001 | 0.855177 |
| P02767 | Transthyretin | Ttr | 7 (7) | 323.31 | 59.9 | 0.025 | -0.45257 |
| P05545 | Serine protease inhibitor A3K | Serpina3k | 30 (10) | 323.31 | 65.9 | <0.001 | -0.76091 |
| Q6MG79 | Complement C4 | C4a | 79 (4) | 323.31 | 60.7 | 0.020 | -0.4852 |
| P08932 | T-kininogen 2 | Kng1l1 | 28 (8) | 323.31 | 62.1 | 0.008 | -0.33983 |
| P10959 | Carboxylesterase 1C | Ces1c | 33 (10) | 323.31 | 65.8 | 0.013 | -0.34625 |
| P12346 | Serotransferrin | Tf | 83 (81) | 323.31 | 71.3 | 0.030 | -0.22207 |
| P14046 | Alpha-1-inhibitor 3 | A1i3 | 112 (4) | 323.31 | 68.7 | 0.002 | 0.366368 |
| P20762 | Ig gamma-2C chain C region |  | 15 (13) | 323.31 | 63.8 | 0.006 | -0.55651 |
| P36953 | Afamin | Afm | 41 (41) | 323.31 | 67.9 | 0.027 | 0.131773 |
| Q01177 | Plasminogen | Plg | 53 (24) | 323.31 | 75 | 0.022 | -0.63233 |
| Q3KR94 | Vitronectin | Vtn | 19 (19) | 323.31 | 50.4 | 0.016 | 0.268395 |
| Q569B3 | Igh-6 protein | Igh-6 | 20 (2) | 323.31 | 39.9 | 0.008 | 0.597809 |
| Q5M7T5 | Serine (Or cysteine) peptidase inhibitor, clade C (Antithrombin), member 1 | Serpinc1 | 30 (11) | 323.31 | 64.1 | 0.025 | -0.35402 |
| Q5M891 | C4b-binding protein alpha chain | C4bpa | 20 (2) | 223.51 | 39.4 | <0.001 | 0.832699 |
| Q68FP1 | Gelsolin | Gsn | 33 (33) | 323.31 | 62.7 | 0.002 | -0.5905 |
| Q6IRS6 | Fetuin-B | Fetub | 24 (24) | 323.31 | 62.1 | 0.004 | -0.56815 |
| Q8CIP8 | Complement component C2 | C2 | 20 (10) | 133.94 | 34.3 | 0.024 | -0.23979 |
| Q6MG90 | Complement C4B (Chido blood group) | C4a | 74 (17) | 323.31 | 57.6 | 0.005 | -0.53623 |
| Q811M5 | Complement component C6 | C6 | 16 (1) | 323.31 | 30.3 | 0.001 | -0.73576 |

*Values of negative fold-change indicate lower protein expression in the obese group when compared to control, while positive values indicate higher expression in the obese group.

**Table S4** Gene ontology (GO)-term enrichment analysis performed with the DAVID Bioinformatics Resources 6.8 from the pool of the proteins significantly changed in the plasma of control and obese rats (Bonferroni statistic).

| **Category** | **Term** | **Count** | **%** | **Fold Enrichment** | **p-value** |
| --- | --- | --- | --- | --- | --- |
| GOTERM_BP_DIRECT | GO:0010951~negative regulation of endopeptidase activity | 15 | 36.56 | 44.43 | 2.66E-17 |
| GOTERM_BP_DIRECT | GO:0006956~complement activation | 6 | 14.63 | 175.35 | 5.32E-08 |
| GOTERM_BP_DIRECT | GO:0006958~complement activation, classical pathway | 7 | 17.07 | 82.94 | 7.72E-08 |
| GOTERM_BP_DIRECT | GO:0006953~acute-phase response | 6 | 14.63 | 69.22 | 8.57E-06 |
| GOTERM_BP_DIRECT | GO:0045087~innate immune response | 7 | 17.07 | 11.76 | 0.009 |
| GOTERM_BP_DIRECT | GO:0006954~inflammatory response | 7 | 17.07 | 10.33 | 0.02 |
| GOTERM_BP_DIRECT | GO:0034014~response to triglyceride | 3 | 7.32 | 263.03 | 0.02 |
| GOTERM_BP_DIRECT | GO:0051918~negative regulation of fibrinolysis | 3 | 7.32 | 187.88 | 0.04 |

**Table S5** List of significantly changed proteins in the left ventricles of hearts from AWL (after weight loss) and ContA (control) groups identified by LC-MS/MS (Student’s T test) (n=8).

| Accession number | Protein name | Gene name | Peptide count (unique peptides) | Confidence score | | Sequence coverage (%) | p-value | Fold change*** |
| --- | --- | --- | --- | --- | --- | --- | --- | --- |
| Q5M819 | Phosphoserine phosphatase | Psph | 5 (5) | | 4.2269 | 17.8 | <0.001 | 20.4 |
| Q00981 | Ubiquitin carboxyl-terminal hydrolase isozyme L1 | Uchl1 | 17 (17) | | 323.31 | 83.4 | 0.002 | -15.9 |
| Q75Q41 | Mitochondrial import receptor subunit TOM22 homolog | Tomm22 | 5 (5) | | 129.03 | 62 | 0.006 | 16.4 |
| Q7TMZ9 | Cardiac titin N2B isoform | N/A | 38 (31) | | 321.86 | 41.6 | 0.011 | 15.2 |
| P12007 | Isovaleryl-CoA dehydrogenase, mitochondrial | Ivd | 21 (21) | | 323.31 | 68.4 | 0.013 | -0.4 |
| P51886 | Lumican | Lum | 12 (12) | | 180.03 | 41.7 | 0.014 | 0.6 |
| Q5XIG4 | OCIA domain-containing protein 1 | Ociad1 | 10 (10) | | 32.111 | 46.2 | 0.019 | 13.7 |
| P39069 | Adenylate kinase isoenzyme 1 | Ak1 | 18 (18) | | 323.31 | 80.4 | 0.019 | -0.6 |
| B5DEF6 | Acyl-CoA dehydrogenase family member 11 | Acad10 | 22 (22) | | 81.795 | 22.3 | 0.021 | 13.9 |
| P45592 | Cofilin-1 | Cfl1 | 24 (20) | | 323.31 | 83.1 | 0.023 | -0.4 |
| G3V7C6 | Tubulin beta-4B chain | Tubb4b | 30 (1) | | 323.31 | 78.1 | 0.035 | -0.5 |
| Q75Q39 | Mitochondrial import receptor subunit TOM70 | Tomm70a | 24 (24) | | 323.31 | 50.2 | 0.039 | 12.0 |
| M0RBF1 | Complement C3 | C3 | 96 (96) | | 323.31 | 73.7 | 0.044 | -0.6 |
| G3V8U8 | Branched-chain-amino-acid aminotransferase | Bcat2 | 15 (15) | | 275.31 | 39.7 | 0.049 | -0.4 |

***Values of negative fold-change indicate lower protein expression in the AWL group when compared to ContA, while positive values indicate higher expression in the AWL group.

**Table S6** List of significantly changed proteins in the plasma of AWL (after weight loss) and ContA (control) groups identified by LC-MS/MS (Student’s T test) (n=9).

| Accession number | Protein name | Gene name | Peptide count (unique peptides) | Confidence score | Sequence coverage (%) | p-value | Fold change*** |
| --- | --- | --- | --- | --- | --- | --- | --- |
| A0A1K0FUB2 | Myoglobin | Mb | 11 (11) | 185.31 | 68.2 | <0.001 | 31.13 |
| E9PSU8 | Ig-like domain-containing protein | N/A | 3 (2) | 72.329 | 31.4 | 0.009 | 1.94 |
| B2RZC1 | Retinol-binding protein 4 | Rbp4 | 10 (10) | 226.59 | 52.7 | 0.048 | 1.46 |
| D3ZBS2 | Inter-alpha-trypsin inhibitor heavy chain H3 | Itih3 | 34 (34) | 323.31 | 47.6 | 0.011 | 0.9 |
| Q5EBC0 | Inter alpha-trypsin inhibitor, heavy chain 4 | Itih4 | 50 (50) | 323.31 | 67 | 0.031 | 0.9 |
| Q5M7T5 | Serine (Or cysteine) peptidase inhibitor, clade C (Antithrombin), member 1 | Serpinc1 | 41 (29) | 323.31 | 70.1 | 0.007 | 0.87 |
| M0RBF1 | Complement C3 | C3 | 139 (11) | 323.31 | 87.9 | 0.001 | 0.86 |
| P20059 | Hemopexin | Hpx | 37 (37) | 323.31 | 68.7 | 0.011 | 0.86 |
| A0A096P6L9 | Complement C5 | C5 | 77 (71) | 323.31 | 55.1 | 0.017 | 0.85 |
| Q5BKC4 | Complement component C9 | C9 | 31 (31) | 323.31 | 63.5 | 0.012 | 0.84 |
| Q6MG74 | B-factor, properdin | Cfb | 46 (3) | 104.57 | 61.2 | 0.016 | 0.8 |
| P31211 | Corticosteroid-binding globulin | Serpina6 | 14 (14) | 307.8 | 41.9 | 0.005 | 0.79 |
| A0A0G2K8K3 | Heparin cofactor 2 | Serpind1 | 16 (16) | 161.48 | 34.2 | 0.001 | 0.75 |
| P02767 | Transthyretin | Ttr | 7 (7) | 113.44 | 62.6 | 0.004 | 0.65 |

***Values of fold-change <1 indicate lower protein expression in the AWL group when compared to ContA, while values>1 indicate higher expression in the AWL group.

| Table S7 Concentration of triglycerides and cholesterol in sera collected from rats after weight loss  (AWL) and control individuals (ContA) (Student’s T-test) (n=6). | | | | |
| --- | --- | --- | --- | --- |
| Parameter | Group | | Significance |  |
|  | ContrA | AWL |  |  |
| Blood parameters |  |  |  |  |
| Triglicerydes (mg/dL) | 105.7 (±17.7) | 84.67 (±19.89) | p=0.082 |  |
| Total cholesterol (mg/dL) | 92 (±6.8) | 92.5 (±12.58) | P=0.93 |  |
| LDL (mg/dL) | 31.5 (±0.84) | 34.33 (±5.99) | p=0.54 |  |
| HDL (mg/dL) | 55.6 (±4.26) | 55 (±5.67) | p=0.84 |  |
| Total cholesterol/HDL ratio | 1.66 (±0.056) | 1.68 (±0.07) | p=0.49 |  |

# **Supplementary figures (S1-3):**

Figures S1-S2 Masson Trichrome staining. S3 H&E staining.

**Figure S2** The left ventricles stained by Masson Trichrome techniques. Example photos of interstitial (A-AWL, B-ContA) and perivascular fibrosis (C-AWL, D-ContA). The collagen fibers are stained blue (magnification ×400).


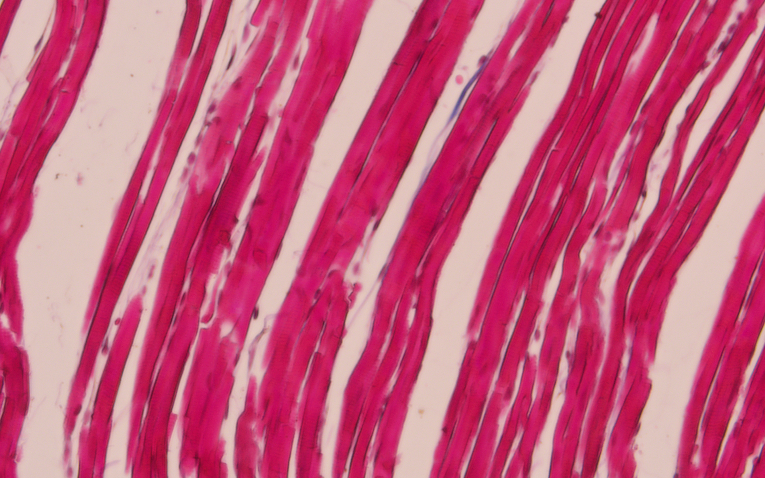

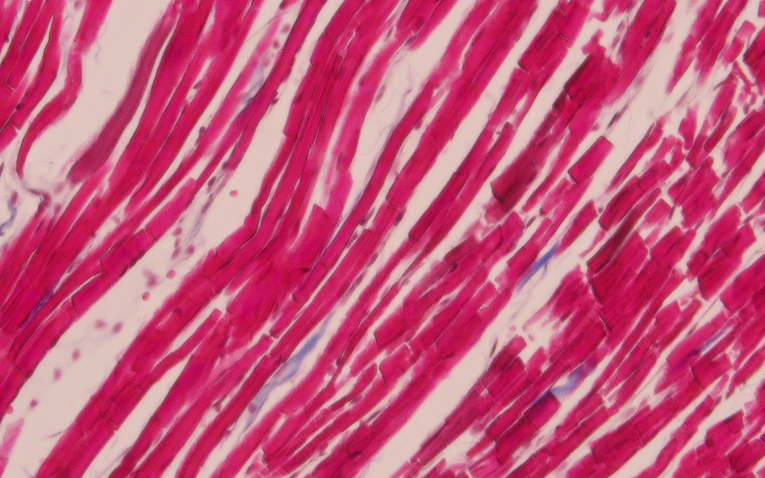

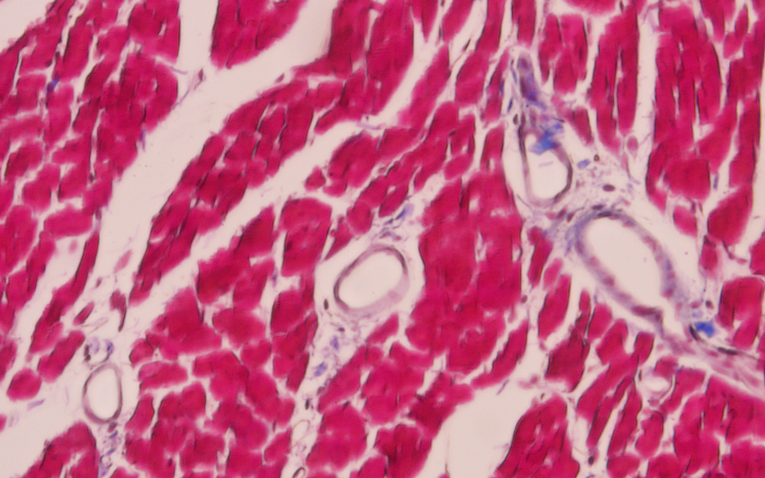

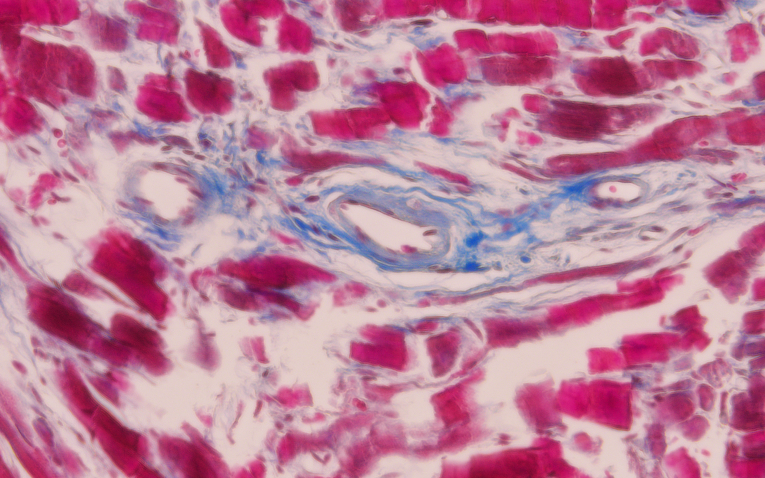


**D**

**B**

**A**

**C**


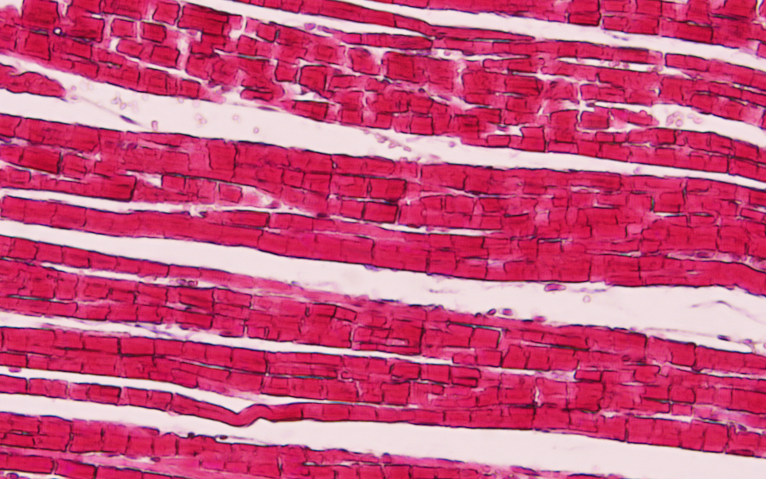

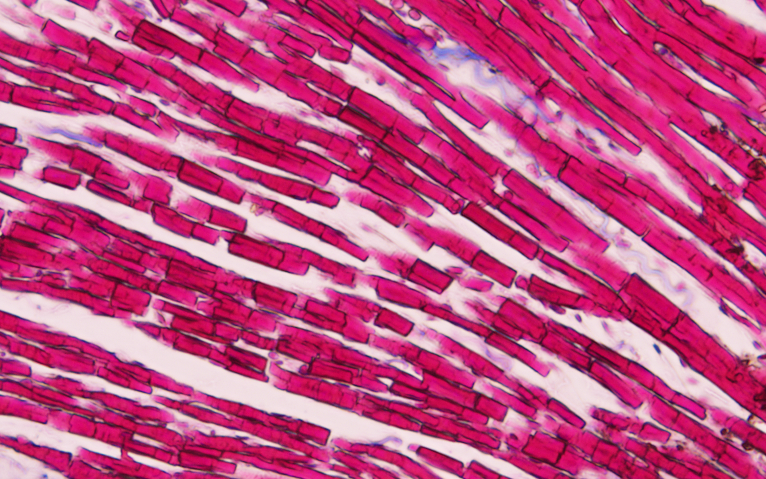

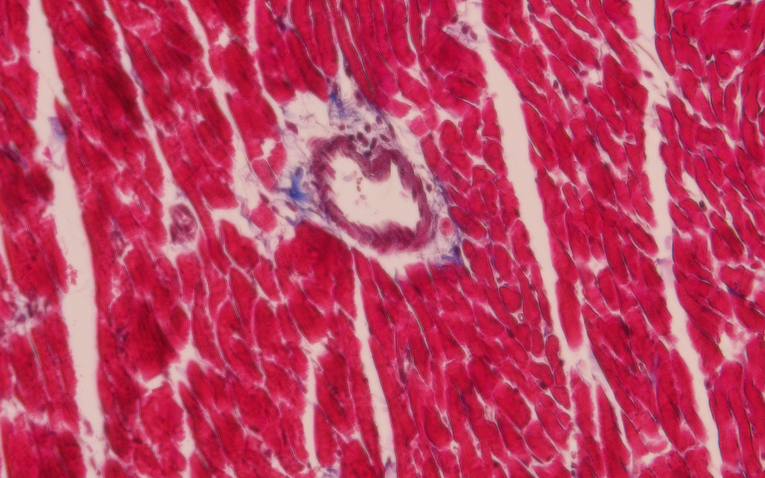

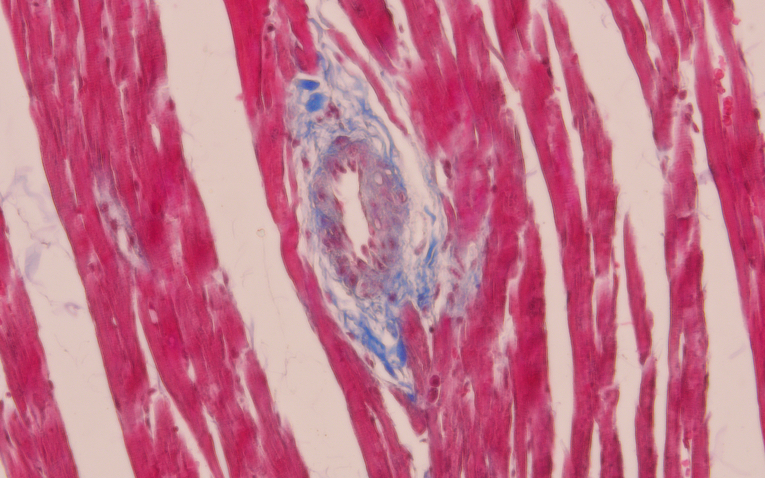


**A**

**B**

**D**

**C**

**Figure S1** The left ventricles stained by Masson Trichrome techniques. Example photos of interstitial (A-obese, B-ContO) and perivascular fibrosis (C-obese, D-ContO). The collagen fibers are stained blue (magnification ×400).


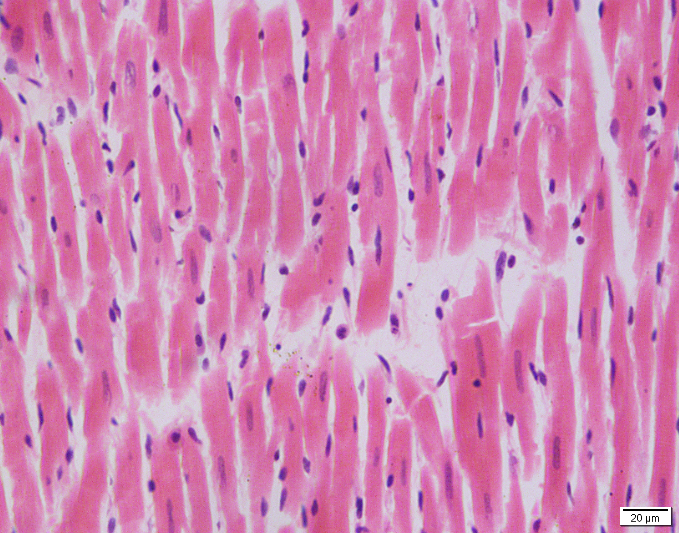


**A**


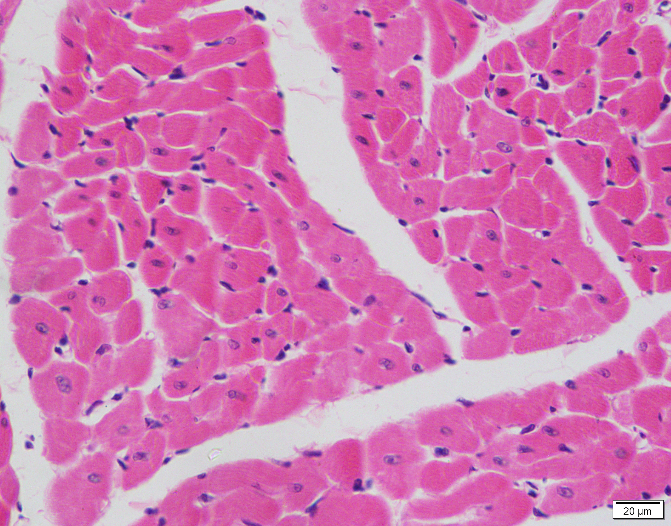


**B**


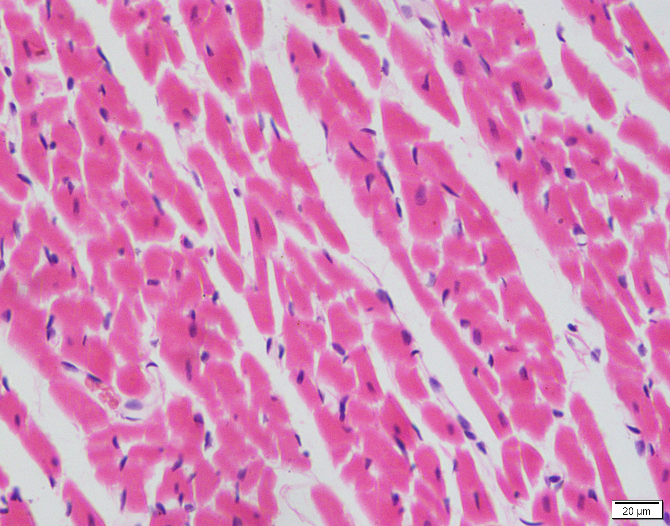

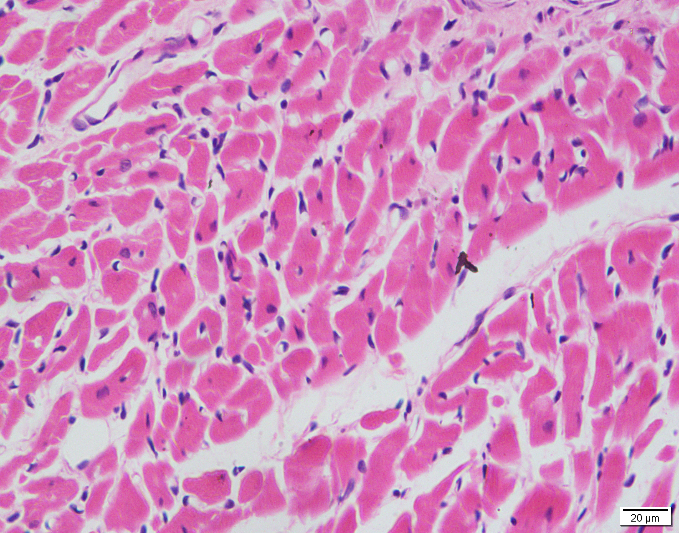


**D**

**C**

**Figure S3** The left ventricles of the heart stained by H&E techniques. Example photos of A-obese, B-ContO, C-AWL, 4-ContA groups (magnification ×400).
